# Wheat MYOSIN-RESEMBLING CHLOROPLAST PROTEIN controls B-type starch granule initiation timing during endosperm development

**DOI:** 10.1093/plphys/kiae429

**Published:** 2024-08-19

**Authors:** Jiawen Chen, Yi Chen, Alexander Watson-Lazowski, Erica Hawkins, J Elaine Barclay, Brendan Fahy, Robin Denley Bowers, Kendall Corbin, Frederick J Warren, Andreas Blennow, Cristobal Uauy, David Seung

**Affiliations:** John Innes Centre, Norwich Research Park, Norwich NR4 7UH, UK; John Innes Centre, Norwich Research Park, Norwich NR4 7UH, UK; John Innes Centre, Norwich Research Park, Norwich NR4 7UH, UK; Harper Adams University, Newport TF10 8NB, UK; John Innes Centre, Norwich Research Park, Norwich NR4 7UH, UK; John Innes Centre, Norwich Research Park, Norwich NR4 7UH, UK; John Innes Centre, Norwich Research Park, Norwich NR4 7UH, UK; John Innes Centre, Norwich Research Park, Norwich NR4 7UH, UK; Quadram Institute, Norwich Research Park, Norwich NR4 7UQ, UK; Department of Horticulture, College of Agriculture, Food and Environment, University of Kentucky, Lexington, KY 40546-0312, USA; Quadram Institute, Norwich Research Park, Norwich NR4 7UQ, UK; Department of Plant and Environmental Science, University of Copenhagen, Thorvaldsensvej 40, Frederiksberg, Copenhagen 1871, Denmark; John Innes Centre, Norwich Research Park, Norwich NR4 7UH, UK; John Innes Centre, Norwich Research Park, Norwich NR4 7UH, UK

## Abstract

Molecular factors that contribute to the diverse spatial and temporal patterns of starch granule initiation between species and organs are poorly understood. Wheat (*Triticum* sp.) endosperm contains both large A-type granules initiated during early grain development and small B-type granules that initiate about 10 to 15 days later. Here, we identify that the MYOSIN-RESEMBLING CHLOROPLAST PROTEIN (MRC) is required for the correct timing of B-type granule initiation in wheat endosperm during grain development. MRC is expressed in the endosperm exclusively in early grain development, before B-type granule initiation. We isolated three independent TILLING mutants of tetraploid wheat (*Triticum turgidum* cv. ‘Kronos’) with premature stop or missense mutations in the A-genome homeolog, which we showed to be the only active homeolog in tetraploid wheat due to a disruption of the B-genome homeolog. The *mrc* mutants had significantly smaller A-type granules and a higher relative volume of B-type granules in the endosperm than the wild type. Whereas B-type granules initiated 15 to 20 days post-anthesis (dpa) in the wild type, they appeared as early as 10 dpa in the *mrc-1* mutant. These results suggest a temporal role for MRC in repressing B-type granule initiation, providing insight into how the distinct biochemical mechanisms that control A- and B-type granule initiation are regulated. This role of MRC in the wheat endosperm is distinct from the previously described role of Arabidopsis (*Arabidopsis thaliana*) MRC in promoting granule initiation in leaves, providing an example of functional diversification among granule initiation proteins.

## Teaser

A starch granule initiation protein in wheat expressed during early endosperm development represses B-type granule initiation.

## Introduction

Starch is the major storage carbohydrate in leaves, seeds, and storage organs of most plants. It is synthesized in plastids as insoluble granules composed of two glucose polymers: amylopectin and amylose. Amylopectin is a branched polymer with linear α-1,4-glucan chains and α-1,6-branch points, and forms the semi-crystalline starch granule matrix—typically constituting 70% to 90% w/w of starch (Smith and Zeeman 2020). Amylose constitutes 10% to 30% w/w of starch and is composed primarily of long α-1,4-linked linear chains (Seung 2020). The biosynthesis of the starch polymers is relatively well understood at the molecular level, and is generally conserved between leaf and storage starches (Smith and Zeeman 2020). However, we are only beginning to understand how starch granule formation is initiated, and the factors underpinning the vast diversity in granule initiation patterns observed between different organs and species (Tetlow and Emes 2017; Seung and Smith 2019; Chen et al. 2021).

A prime example of diverse granule initiation patterns between species can be observed in the seed endosperms of grasses (Matsushima et al. 2013). Species of the Triticeae, including important cereal crops such as wheat (*Triticum* sp.), barley (*Hordeum vulgare*) and rye (*Secale cereale*), have a unique bimodal size distribution of starch granules in the grain endosperm—containing large flattened A-type granules (20 to 30 *µ*m in diameter) and small round B-type granules (2 to 7 *µ*m in diameter) (Howard et al. 2011). The initiation of these two different types of granules is both spatially and temporally separated: A-type granules initiate in amyloplasts as early as 4 days post-anthesis (dpa), whereas B-type granules initiate 10 to 15 days after the A-type granules (i.e. around 15 to 20 dpa) and at least partly within stromules that emanate from the amyloplast (Parker 1985; Bechtel et al. 1990; Langeveld et al. 2000; Howard et al. 2011). In wheat, the small B-type granules typically make up more than 90% of granules by number, but less than 30% by volume (Lindeboom et al. 2004; Kamble et al. 2023). This is distinct from most other grass species, which produce “compound” starch granules—where multiple granules initiate early during grain development in each amyloplast and eventually fuse (e.g. in rice) (Matsushima et al. 2013).

Our recent work has identified proteins that are important for the initiation of bimodal starch granules in wheat. STARCH SYNTHASE 4 (SS4) is required for normal A-type granule formation in wheat, as in its absence, compound granules form in place of most A-type granules (Hawkins et al. 2021). B-GRANULE CONTENT1 (BGC1) has a dose-dependent effect on granule initiation in wheat, where partial reductions in gene dosage can almost eliminate B-type granules without affecting A-type granule formation. However, complete knockout of gene function also causes defective A-type granule formation, including the formation of some compound granules that arise from multiple initiations (Howard et al. 2011; Chia et al. 2020; Saccomanno et al. 2022). BGC1 was characterized in wheat as well as in *Aegilops*, and is orthologous to FLOURY ENDOSPERM6 (FLO6) in barley and rice and PROTEIN TARGETING TO STARCH 2 (PTST2) in Arabidopsis (*Arabidopsis thaliana*) and *Brachypodium* (Peng et al. 2014; Seung et al. 2017; Saito et al. 2018; Chia et al. 2020; Watson-Lazowski et al. 2022). The increased number of initiations per amyloplast that led to compound granule formation in the *ss4* and *bgc1* mutants was unexpected since in Arabidopsis leaves, both SS4 and PTST2 promote granule initiation, and mutants lacking either protein have reduced numbers of starch granules per chloroplast (Roldán et al. 2007; Seung et al. 2017). Together with BGC1, the plastidial α-glucan phosphorylase (PHS1) specifically promotes B-type granule initiation in the wheat endosperm, and is not required for A-type granule initiation (Kamble et al. 2023). This role for PHS1 in B-type granule initiation again stands in contrast to Arabidopsis leaves, where loss of PHS1 alone does not affect numbers of starch granules (Malinova et al. 2014; Kamble et al. 2023). These observations suggest that the proteins involved in granule initiation are to some extent conserved between species and organs, but they can act differently depending on the patterns of granule initiation in the species/tissue.

To gain further insight into such differences, we explored the function of MYOSIN-RESEMBLING CHLOROPLAST PROTEIN (MRC, also known as PROTEIN INVOLVED IN STARCH INITIATION, PII1) in amyloplasts of the wheat endosperm. MRC is a long coiled-coil protein that promotes granule initiation in Arabidopsis leaves, as most chloroplasts in the *mrc* mutant contain only a single granule (Seung et al. 2018; Vandromme et al. 2019). The exact function of MRC is unknown, but it is proposed to act via an interaction with SS4 and PTST2 (Seung et al. 2018; Vandromme et al. 2019). We discovered a distinct role for MRC in the temporal control of B-type granule initiation in the wheat endosperm. MRC is expressed at the early stages of grain development, and wheat *mrc* mutants have severe alterations in the starch granule size distribution relative to the wild type, with smaller A-type granules and a higher relative volume of B-type granules. We demonstrate that this phenotype arises from the early initiation of B-type granules in the mutant, suggesting MRC represses B-type granule initiation during early grain development. This temporal role of MRC in the wheat endosperm demonstrates how the function of granule initiation proteins can be adapted to mediate specific patterns of granule initiations among different species/tissues.

## Results

### The wheat orthologs of MRC are encoded on chromosomes 6A and 6D

**Figure 1.**
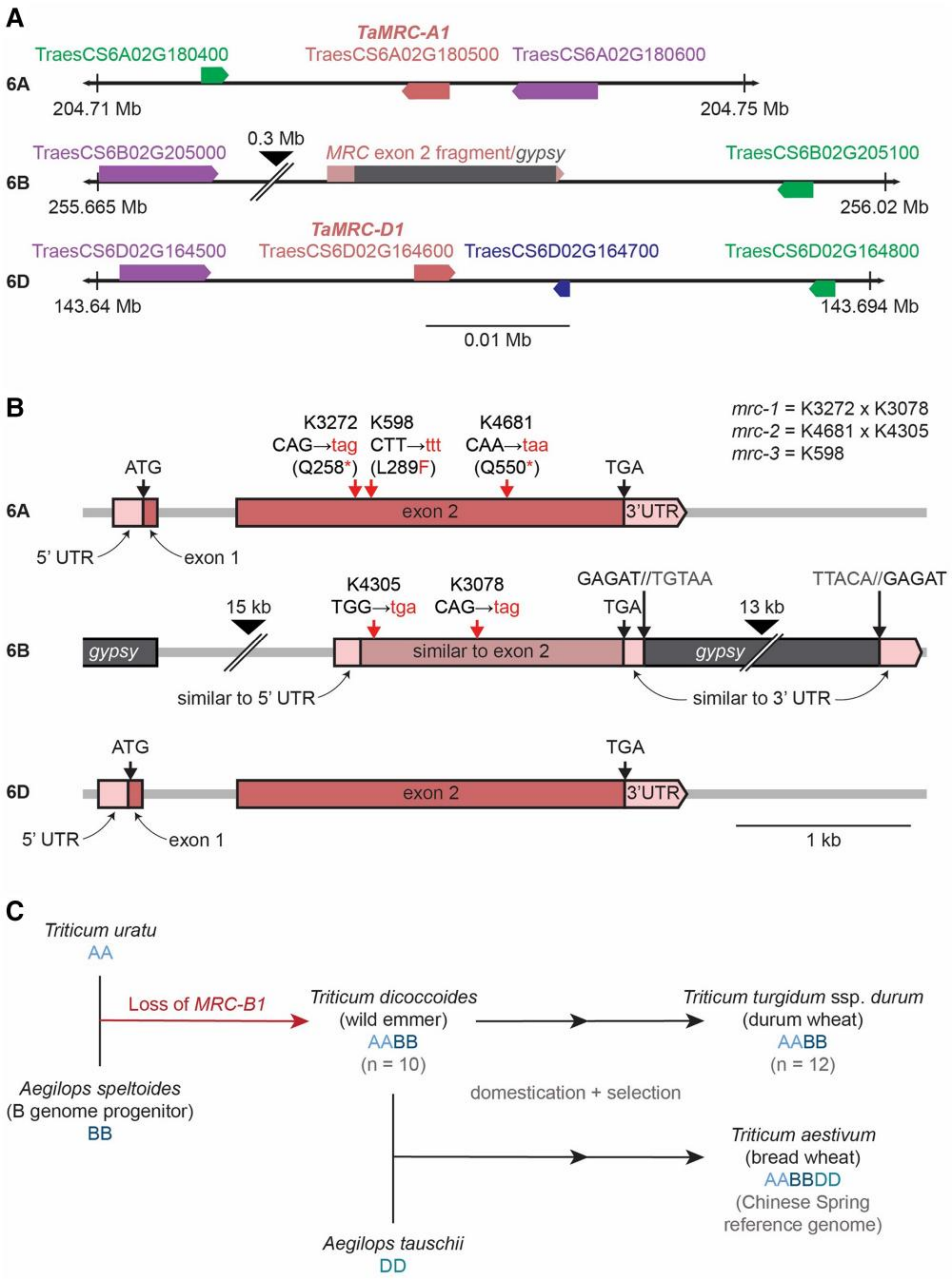
*MRC* homeologs in wheat are encoded on chromosomes 6A and 6D, with a disruption of the 6B homeolog. **A)** Location of *TaMRC* homeologs on chromosome 6A and 6D. The *TaMRC* homeologs (TraesCS6A02G180500 and TraesCS6D02G164600) are shown with the neighbouring genes, cytochrome P450 family protein (TraesCS6A02G180400, TraesCS6B02G205100, TraesCS6D02G164800), respiratory burst oxidase homolog (TraesCS6A02G180600, TraesCS6B02G205000, TraesCS6D02G164500), and an uncharacterized protein (TraesCS6D02G164700). Arrowheads on the boxes indicate direction of transcription. The syntenic region on chromosome 6B has a large insertion, depicted with a black arrowhead. The diagram is drawn to scale, and chromosome coordinates of the region are indicated. **B)** Gene models of the *TaMRC-A1* and -*D1* homeologs and 6B pseudogene. Exons are represented with boxes, while lighter boxes represent the 5′ and 3′ UTRs. On the 6B region, areas with sequence similarity to exon 2 and UTRs of *TaMRC-A1* are indicated, as well as the location of *gypsy* retrotransposons. The locations of the mutations in the *mrc* mutants are depicted with red arrows, and the mutated codons/amino acids are shown in red letters. Large black arrowheads show where the sequence has been truncated for illustration—the length of truncated sequence is indicated above. **C)** Summary of species analyzed for the loss of *MRC-B1* during wheat hybridization.

The starch granule initiation protein MRC is highly conserved among land plants (Seung et al. 2018). To determine the role of MRC in wheat, we searched the wheat genome for genes encoding MRC orthologs. We ran a BLASTp search using the amino acid sequence of Arabidopsis MRC (*At*MRC, At4g32190) against the protein sequences from both tetraploid durum wheat (*Triticum turgidum*; Svevo v1.1) (Maccaferri et al. 2019) and hexaploid bread wheat (*Triticum aestivum*; IWGSG Chinese Spring) (Appels et al. 2018) genomes on Ensembl Plants. For hexaploid wheat, the two top protein hits were TraesCS6A02G180500.1 (encoded on chromosome 6A) and TraesCS6D02G164600.1 (encoded on chromosome 6D), which shared 95% sequence identity with each other and were predicted as homeologs on Ensembl. Both genes had a two-exon structure like the Arabidopsis gene (Seung et al. 2018), and were in syntenic positions on the A and D genomes (Fig. 1). Pairwise alignments of *At*MRC and *Ta*MRC show 32% sequence identity and 56% to 57% sequence similarity, depending on the homeolog used in the comparison (Supplementary Table S1). For tetraploid durum wheat, the top protein hit was TRITD6Av1G081580.1 (encoded on chromosome 6A), which was identical in nucleotide and amino acid sequence to TraesCS6A02G180500.1. To determine whether these proteins were true orthologs of *At*MRC, we repeated the phylogenetic analyses of MRC homologs from our previous study (Seung et al. 2018) with the wheat protein sequences included. The 6A and 6D proteins clustered together on the tree, distinctly within the grass clade containing the rice (*Oryza sativa*) and maize (*Zea mays*) sequences (Supplementary Fig. S1A). This confirms that the proteins are the wheat orthologs of MRC. They will hereafter be referred to as *Ta*MRC-A1 (TraesCS6A02G180500) or *Tt*MRC-A1 (TRITD6Av1G081580), and *Ta*MRC-D1 (TraesCS6D02G164600). Notably, we did not find a full gene model for MRC on chromosome 6B, or anywhere else on the B genome, either in the durum or bread wheat genome.

To investigate why no homeolog was detected on chromosome 6B, we looked at the syntenic region of chromosome 6B in Chinese Spring, where there was a stretch of sequence that had homology to exon 2 and the beginning of the 3′UTR (Fig. 1A). Interestingly, around 14 kb downstream of that, there was a region highly similar to the end of the 3′ UTR of *TaMRC-A1*. We looked at the transposable element annotation of the wheat reference genome around the exon 2 fragment (Daron et al. 2014), and a complete *gypsy* retrotransposon was annotated between the two 3′UTR fragments (Fig. 1B). Further, we identified the 5 bp target site duplication (GAGAT, which is part of the 3′UTR) and the inverted terminal repeat (TGTAA and TTACA at the start and end of the retrotransposon, respectively) characteristic of retrotransposon insertions. The distance between the 5′ end of the exon 2 fragment and its upstream neighboring gene (TraesCS6B02G20500, a respiratory burst oxidase homolog) was much larger than the distance between *Ta*MRC and the homeologs of the same neighboring gene on 6A and 6D, indicating a large insertion in this 6B region (Fig. 1A). Indeed, a fragment of another *gypsy* retrotransposon was found ca. 16 kb upstream of the exon 2 fragment. Additionally, there was a sequence with homology to the 5′ UTR of *TaMRC-A1* (85% identity over 243 bp) just 3 bp upstream of the exon 2 fragment, which suggested that a ∼1.3 kbp deletion (based on A-genome distances) removed some of the 5′ UTR, all of exon 1, intron 1, and the start of exon 2 of *TaMRC-B1*. Similar to Chinese Spring, we found identical disruptions in *TaMRC-B1* sequences with retrotransposon insertions and deletions in 10 additional wheat genome assemblies (Walkowiak et al. 2020). Overall, it appears that a deletion and a series of transposon insertions severely disrupted *MRC* on chromosome 6B in bread wheat.

Since a B-genome copy was also absent from durum wheat, it is likely that the disruption of *MRC* on chromosome 6B preceded the second hybridization that resulted in hexaploid wheat. To further investigate when the disruption of *MRC-B1* occurred, we looked for *MRC-B1* in more tetraploid wheat accessions by aligning genome-sequencing reads from *Triticum dicoccoides* (wild emmer) (*n* = 10) and *T. turgidum* ssp. *durum* (pasta wheat) (*n* = 12) against the A and B genomes of Chinese Spring (Zhou et al. 2020) (Supplementary Table S2). The exon 1 deletion and the retrotransposon insertion at the 3′ end were detected in all accessions (except for a few lines that had poor sequencing depth in the region), suggesting that *TaMRC* on 6B was disrupted before or immediately after the hybridization of diploid ancestors carrying the A and B genomes. To distinguish these possibilities, we examined *MRC* in *Aegilops speltoides*, the diploid species thought to be most closely related to the progenitor species of the wheat B genome. We ran a BLASTn search on the genome assembly of the *A. speltoides* accession TS01 using the coding sequence of *TaMRC-A1* (Li et al. 2022) (Supplementary File 1). The top hit was on chromosome 6S (homologous to chromosome 6B in wheat) with intact exons 1 and 2 (97.5% sequence identity), and the translated protein sequence had 96% identity to *Ta*MRC-A1 with BLASTp. Thus, it is likely that *MRC* is intact in *A. speltoides*, suggesting that the loss of the B-homeolog occurred shortly after the hybridization that gave rise to tetraploid wheat (Fig. 1C). It is therefore expected that all tetraploid wheats have 1 *MRC* homeolog (on chromosome 6A), and all hexaploids have 2 (on chromosomes 6A and 6D).

### MRC is expressed early in developing endosperm

**Figure 2.**
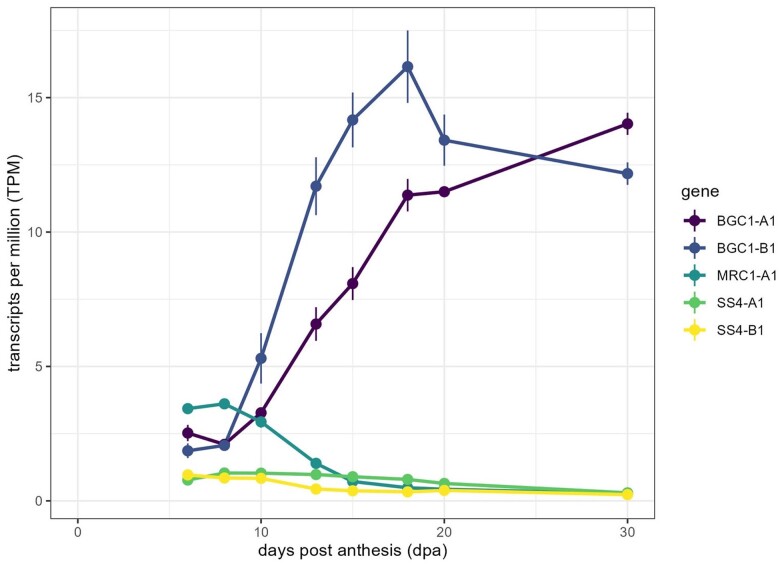
*MRC* is expressed in wheat endosperm during early grain development. Expression of *TtMRC-A1* (TRITD6Av1G081580.1), *TtBGC1-A1*, *TtBGC1-B1*, *TtSS4-A1*, *TtSS4-B1* in the starchy endosperm during grain development of *Triticum turgidum* (variety ‘Kronos’). Data are from an RNAseq experiment described in Chen et al. (2023). Values represent average transcripts per million (TPM) ± SEM from *n* = 3 for all time points.

We examined the expression pattern of *MRC* in the endosperm through grain development using our existing RNAseq dataset on developing endosperms of *T. turgidum* cv. ‘Kronos’ (Chen et al. 2023). ‘Kronos’ is the genetic background of the tetraploid wheat TILLING mutants characterized below. *TtMRC-A1* (TRITD6Av1G081580.1) peaked in expression during early grain development (8 dpa), but strongly decreased in expression between 10 and 20 dpa (Fig. 2). It therefore appears that *MRC* is expressed in the wheat endosperm almost exclusively during early grain development, similar to a number of other granule initiation proteins such as *SS4* (Fig. 2, Chen et al. 2023). In contrast, *BGC1* was expressed in early grain development, but peaked in expression during later grain development, essentially showing an opposite pattern to *MRC*.

### Loss of MRC does not affect the growth of wheat plants, or grain development

To study the function of MRC in wheat, we obtained mutants in durum wheat (*T. turgidum* cv. ‘Kronos’) defective in *MRC*. We used the wheat in silico TILLING mutant resource, which has an EMS-mutagenized population of ‘Kronos’ with exome-capture sequencing data for identification of lines with mutations of interest (Krasileva et al. 2017). We obtained 3 mutants that were likely to cause a loss of function in *TtMRC-6A* (Fig. 1B). The K3272 and K4681 lines contained premature stop codons in place of codons for the 258th and 550th amino acids, respectively. In addition, we obtained a third line (K598) that contained a missense Leu289Phe mutation, which was predicted to be deleterious to protein function by SIFT scoring (Ng and Henikoff 2006). The Leu^289^ residue is highly conserved in all MRC orthologs, and its mutation to a Phe residue is predicted to disrupt coiled-coil formation in the region of the residue (Supplementary Fig. S1, B and C). Since the 6B homeolog of MRC has likely become a pseudogene, we predicted that *TtMRC-A1* would be the only functional *MRC* homeolog in tetraploid wheat. However, to rule out the possibility that the fragment of exon 2 on chromosome 6B affects MRC function, we also obtained the K4305 and K3078 lines which contain two different premature stop codon mutations in the putative reading frame of the exon. We generated the *mrc-1* lines by crossing K3272 and K3078, and the *mrc-2* lines by crossing K4681 and K4305, where we isolated lines homozygous for either the 6A (F_2_  *aa*BB) or 6B (F_2_ AA*bb*) mutation, or both (F_2_  *aabb*). The *mrc-1* double mutant line was backcrossed twice to wild type (WT), and the wild-type segregant (BC_2_F_2_ AABB) and the homozygous double mutant (BC_2_F_2_  *aabb*) were selected. These will be hereafter referred to as BC2 AABB and BC2 *aabb*. No backcrossing was done for the *mrc-2* line. The *mrc-3* line contained the homozygous Leu289Phe missense mutation, and no crossing was done (*aa*BB).

**Figure 3.**
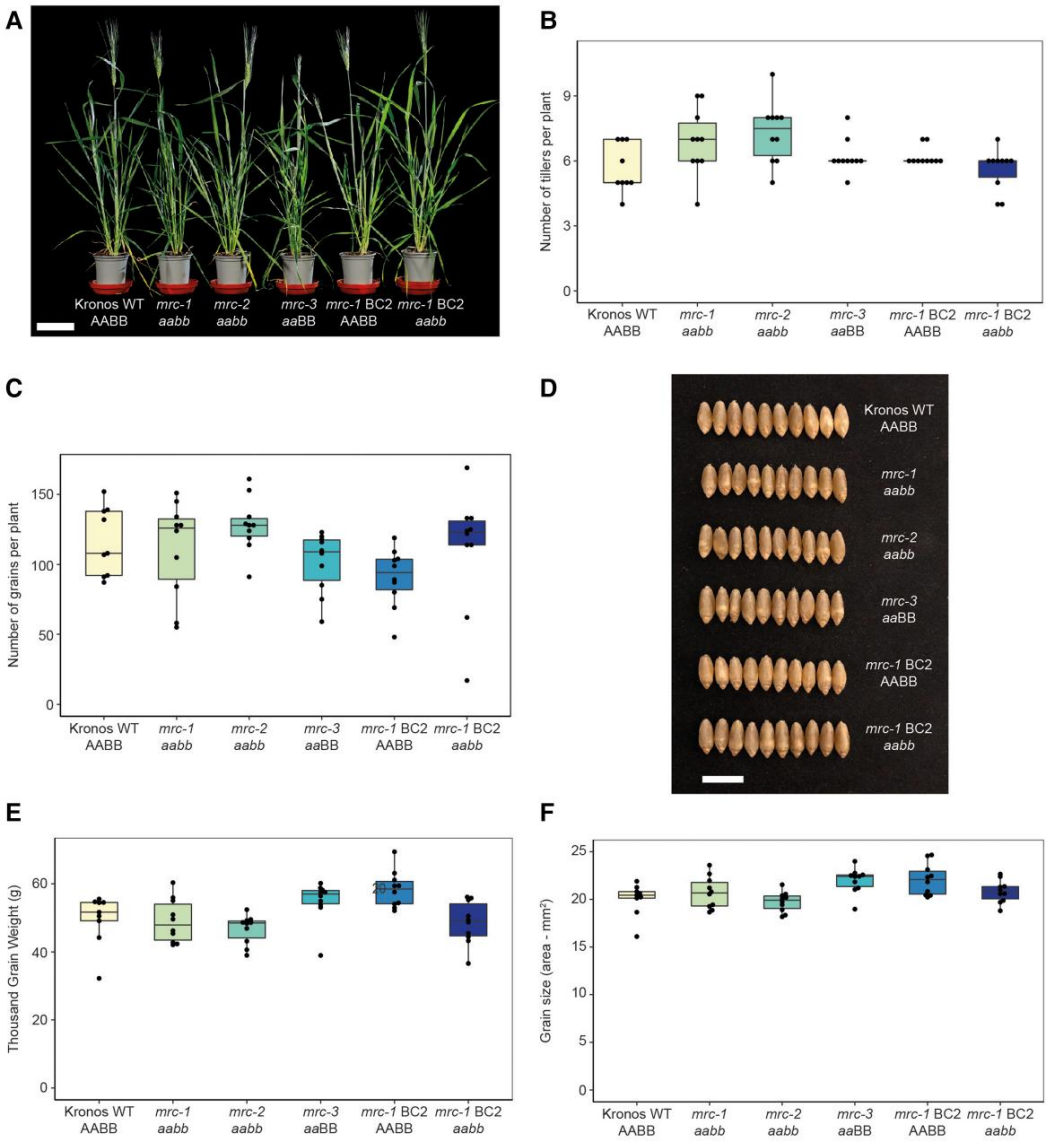
Mutations in *MRC* do not affect plant growth or the number and size of grains. **A)** Photographs of 7-wk-old wild type (WT) and *mrc* mutant plants. Bar = 9 cm. **B)** Number of tillers per plant. **C)** Number of grains per plant. **D)** Photographs of mature grains. Bar = 1 cm. **E)** Thousand grain weight (TGW) of mature grains. **F)** Grain size, measured as the average area of individual mature grains from each plant. For statistical analysis of **B, C, E, F)**, see Supplementary Table S3. For all boxplots, each box encloses the middle 50% of the distribution, the middle line is the median, and the whiskers are the minimum and maximum values within 1.5 × of the interquartile range. Points are measurements from individual plants, with the same group of plants measured for all phenotypes and *n* = 9 to 10 individual plants.

There was no consistent effect of the independent *mrc* mutations on plant growth or grain development under our growth conditions. None of the *mrc* mutant plants appeared different from WT with respect to growth or the number of tillers per plant (Fig. 3, A and B, Supplementary Table S3A). The number of grains per plant also did not differ in the mutants, except for a slight decrease in *mrc-3* and the wild-type segregant (*mrc-1* BC2 AABB) compared to the WT (Fig. 3C, Supplementary Table S3B). The morphology of the mature grains of the mutants was indistinguishable from the WT (Fig. 3D), and there were no differences in thousand grain weight (TGW) and grain size between the WT and any of the three mutants *mrc-1*, *mrc-2*, and *mrc-3*, or between the WT and the backcrossed *mrc-1* (*mrc-1* BC2 *aabb*); but the wild-type segregant (*mrc-1* BC2 AABB) had a slightly higher TGW and grain size compared to WT (Fig. 3, E and F, Supplementary Table S3, C and D). This suggests that some of the background mutations in the wild-type segregant may have affected grain development, but these effects are small.

### Loss of MRC does not strongly affect the total starch content or number in the endosperm

**Figure 4.**
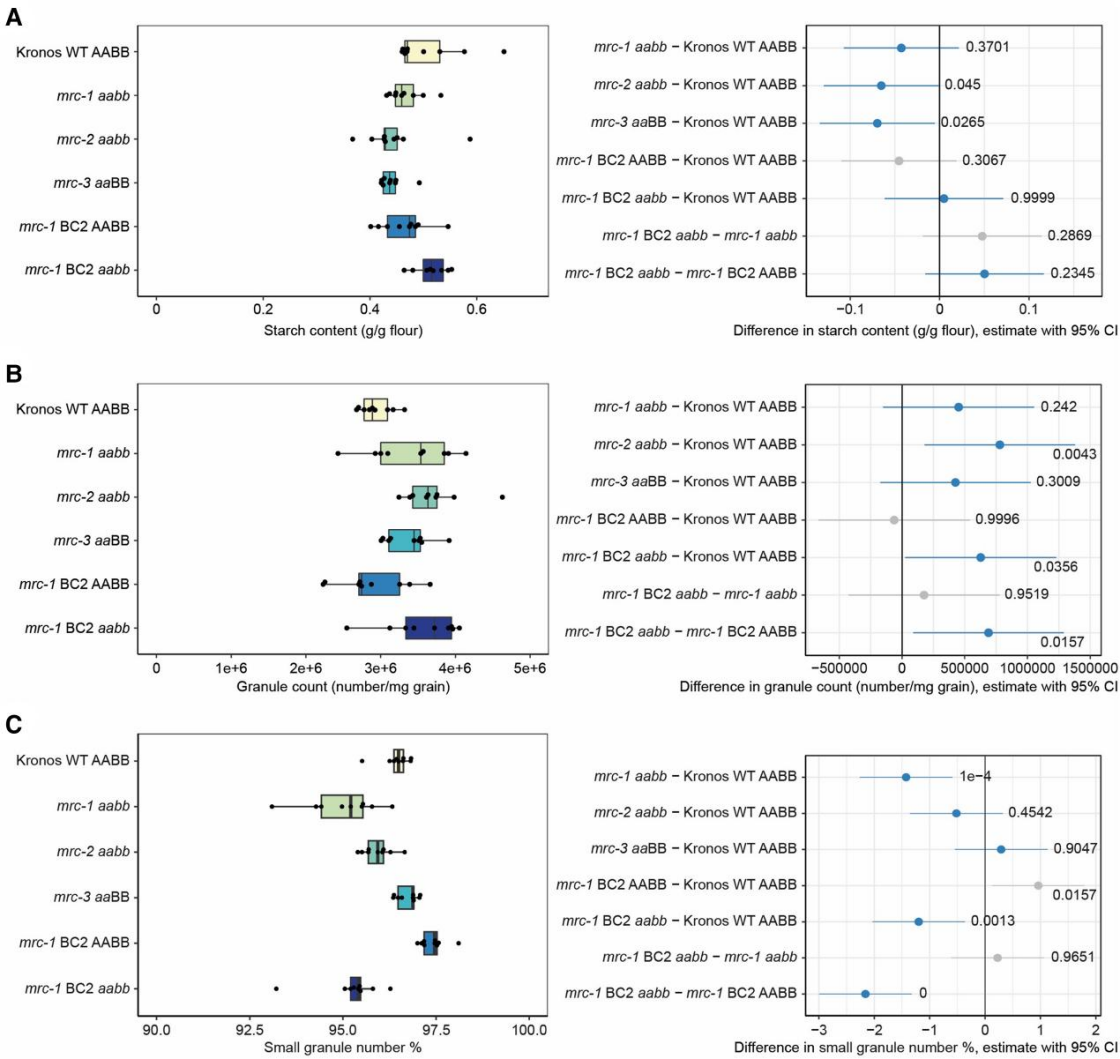
Mutations in *MRC* have variable effects on total starch in mature grains. **A)** Total endosperm starch content in mature grains in the wild type (WT) and *mrc* mutants. **B)** Number of starch granules per mg of grain, counted with a Coulter counter in volumetric mode. **C)** Percentage of small granules (<10 *µ*m) of total granule number. For all plots, points on the boxplots indicate individual plants, with *n* = 8 to 9. Each box encloses the middle 50% of the distribution, the middle line is the median and the whiskers are the minimum and maximum values within 1.5 × of the interquartile range. All statistical analyses were performed using a linear model, with a one-way ANOVA and Tukey post hoc test. Panels on the right indicate differences in means between genotypes from pairwise comparisons based on these models. The difference in means is indicated by a dot, with whiskers showing the 95% confidence interval (CI) of this difference, with the corresponding *P*-value. Gray indicates the WT or *mrc-1* mutant compared to the backcrossed line with the equivalent genotype at the *MRC* loci, and blue indicates all other pairwise comparisons.

To determine the effect of the *mrc* mutations on starch synthesis in grains, we first measured total starch content of mature grains. Starch content was largely similar between WT and the *mrc* mutants. Although some pairwise comparisons showed *P* < 0.05 (WT with *mrc-2* and *mrc-3*), the confidence intervals of the difference in means were still close to zero, suggesting that any effect of the mutations was small (Fig. 4A).

Coulter counter measurements revealed that there were some minor differences between genotypes in the total number of granules relative to grain weight (granules/mg grain) (Fig. 4B) but overall, there was not a strong effect of loss of MRC. The backcrossed *mrc-1* mutant had more starch granules per unit grain weight than both the WT and the wild-type segregant, but the non-backcrossed *mrc-1* mutant was not significantly different to the WT. The *mrc-2* mutant also had relatively more granules than the WT, but *mrc-3* did not. We also calculated the percentage of the number of small starch granules that had a less than 10 *μ*m diameter, which includes mostly B-type granules but also some smaller A-type granules (Chia et al. 2020; Kamble et al. 2023). There was a small but significant decrease in the number of small granules in all the *mrc-1* mutants compared to WT lines (less than 3% difference), but no difference for *mrc-2* and *mrc-3* (Fig. 4C). Overall, mutation of MRC does not seem to have a strong or consistent effect on either the overall granule number (Fig. 4B) or the proportion of the number of small granules (Fig. 4C).

### Loss of MRC greatly alters starch granule size distributions in the endosperm

**Figure 5.**
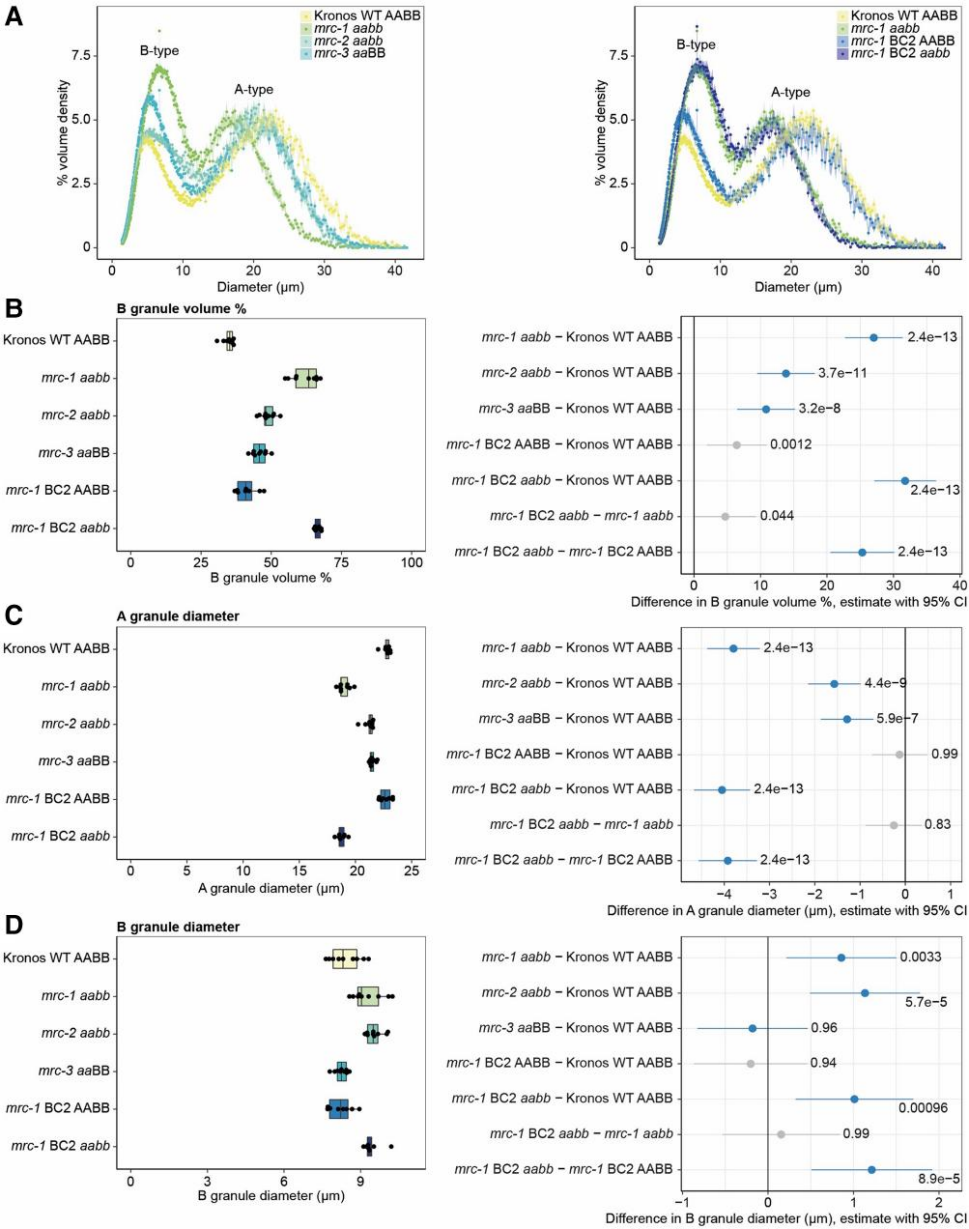
Endosperm starch in mature grains of *mrc* mutants has altered granule size distribution. **A)** Coulter counter traces (measured in volumetric mode) with evenly binned *x* axes show a bimodal distribution of granule sizes by volume percentage from purified wheat endosperm starch. Data points are mean values from 9 individual plants of each genotype (3 grains from each plant), with the standard error of the mean shown as a shaded ribbon. All data from both panels were collected in the same experiment. Wild type (WT) and *mrc-1* in the left and right panels are the same data, shown for ease of comparison with the different *mrc* mutants (left) or *mrc-1* backcrossed (right) lines. **B** to **D)** Mean B-type granule percentage volume **B)**, A-type granule diameter **C)**, and B-type granule diameter **D)** values extracted from bimodal log-normal distribution curves fitted to Coulter counter traces of individual plants. Extracted values and boxplots are shown on the left, where points indicate mean values from individual plants (*n =* 9). Each box encloses the middle 50% of the distribution, the middle line is the median, and the whiskers are the minimum and maximum values within 1.5× of the interquartile range. All statistical analyses were performed using linear models for each panel, with a one-way ANOVA and Tukey post hoc test. Panels on the right indicate differences in means between genotypes from pairwise comparisons based on these models. The difference in means is indicated by a dot, with whiskers showing the 95% confidence interval (CI) of this difference, with the corresponding *P*-value. Gray indicates the WT or *mrc-1* mutant compared to the backcrossed line with the equivalent genotype at the *MRC* loci, and blue indicates all other pairwise comparisons.

In addition to counting the number of granules (Fig. 4), the Coulter counter measures the volume of starch granules, which can be used to generate volumetric granule size distributions by plotting the percentage of starch volume in each size bin (binned by the particle diameter calculated assuming spherical shape) relative to the total volume of starch measured. We observed clear bimodal distributions for all genotypes, with a peak corresponding to A-type granules (∼18 to 25 *µ*m) and a peak corresponding to B-type granules (∼3 to 10 *µ*m) (Fig. 5A). Even though the percentage of small granules by number was similar between the mutants and WT (Fig. 4C), the volumetric size distribution profiles in the mutants were very different from the WT, with the peak corresponding to B-type granules being more prominent in the mutants (Fig. 5A). The increased proportion of B-type granules by volume, in the absence of changes in their proportion by number (Fig. 4C), suggests that the altered volumetric size distributions are primarily driven by changes in granule size. The profiles of the backcrossed *mrc-1* lines looked similar to their non-backcrossed equivalents.

We fitted a bimodal log-normal distribution curve to the profiles of each sample to estimate the total volume percentage of B-type granules and the mean sizes of A- and B-type granules. Comparing the means of these extracted values between genotypes showed a higher B-type granule percentage (by volume) for all 3 mutants (*mrc-1*, *mrc-2*, and *mrc-3*) compared to the WT (Fig. 5B). The strongest increase was seen for *mrc-1*, and this increase was consistent when comparing the double backcrossed *mrc-1* (*mrc-1* BC2 *aabb*) with WT and the wild-type segregant (*mrc-1* BC2 AABB). There was a small increase in the volume percentage of B-type granules in the wild-type segregant compared to the WT, but the difference was much smaller than between the other genotypes. The higher B-type granule volume percentage in *mrc* mutants is most likely due to differences in granule size rather than number, considering there were no major differences in total granule number or proportion of the number of small granules (Fig. 4, B and C). The mean B-type granule size was indeed larger than WT for *mrc-1* (and *mrc-1* BC2 *aabb*) and *mrc-2*, but not *mrc-3* (Fig. 5D). However, the mean A-type granule diameter was smaller for all three mutants than for WT (Fig. 5C). Thus, the increased proportion of B-type granule volume in the mutants is likely due to a combination of smaller A-type granules and larger B-type granules.

**Figure 6.**
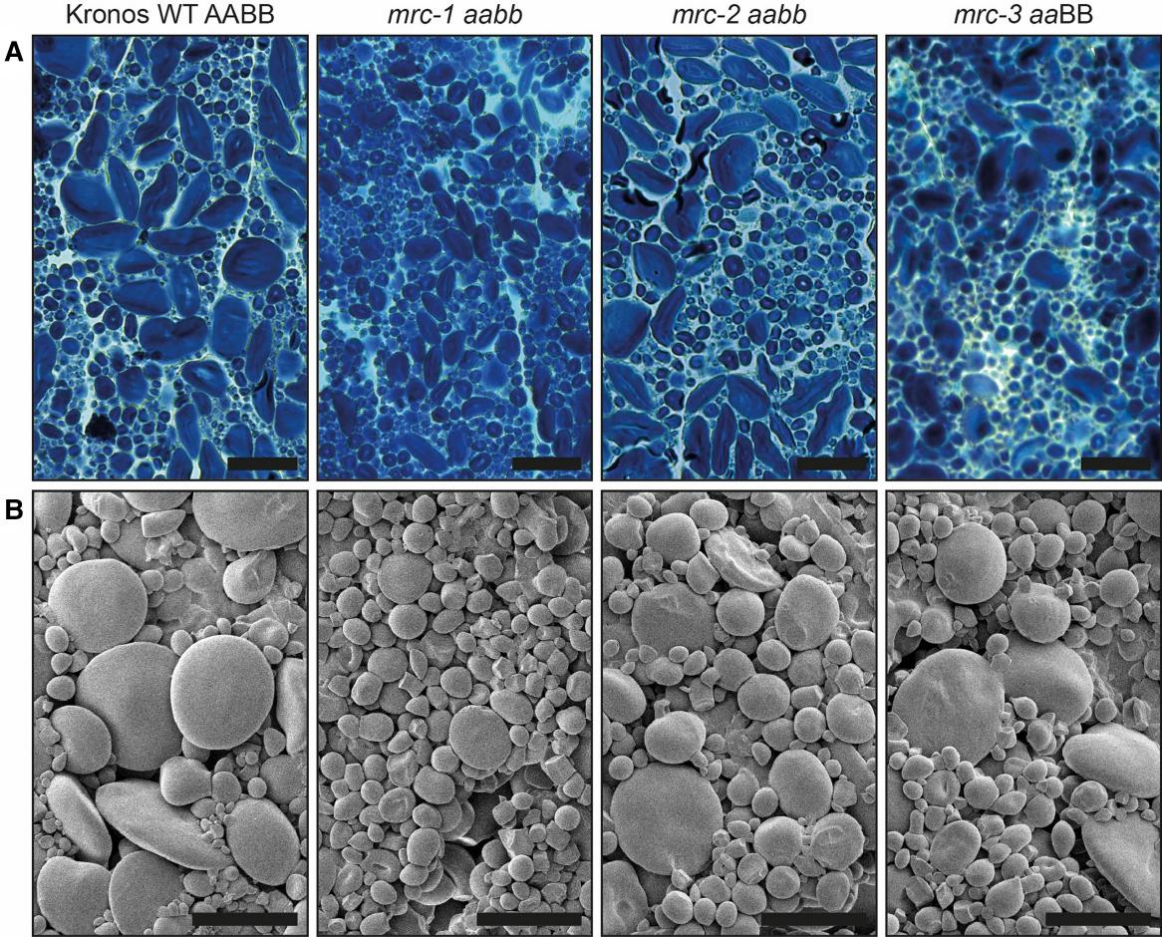
Endosperm starch granules of mature grains in 3 wheat *mrc* mutants are similar in shape compared to wild type (WT). **A)** Thin sections of mature endosperm tissue were stained with Lugol's solution and imaged using light microscopy. Bar = 40 *µ*m. **B)** Purified endosperm starch granules were observed using scanning electron microscopy (SEM). Bar = 20 *µ*m.

We also explored whether the *mrc* mutations affected starch granule shape. Examination of iodine-stained thin sections of mature grains using light microscopy showed that, like the WT, all mutants had flattened A-type and round B-type granules (Fig. 6A). Similarly, no defects in A- or B-type granule shape were observed in the mutants using scanning electron microscopy (SEM) (Fig. 6B). Starch polymer structure and granule composition were not affected by loss of functional MRC. The *mrc-1* mutant, with the strongest alteration in granule size distribution, had normal amylopectin structure and amylose content (Supplementary Fig. S2).

We sought experimental evidence about the contribution of the fragment of exon 2 on chromosome 6B to the observed differences in granule size distribution between genotypes (Supplementary Fig. S3). Quantification of starch granule size distribution in the full set of homozygous genotypes resulting from the crosses that yielded the *mrc-1* and *mrc-2* mutants (indicated as *aa*BB [6A mutant], AA*bb* [6B mutant], and *aabb* [6A and 6B double mutant]) showed that WT and AA*bb* genotypes had identical granule distributions. The *aa*BB and *aabb* had different distributions from the WT but were identical to each other. These data showed that the fragment of exon 2 on chromosome 6B has no influence on granule size distribution. They are consistent with the suggestion that this is a pseudogene, and hence that the 6A copy of *MRC* is the only functional homeolog in tetraploid wheat.

Overall, these data suggest that MRC is required for the normal size distribution of starch granules in wheat endosperm. In tetraploid wheat, mutants lacking in the 6A copy of *MRC* consistently had a higher relative volume of B-type granules in the endosperm than the WT, and smaller A-type granules. This change in granule size distribution occurred without accompanying changes in total starch content, starch granule shape, amylose content, or amylopectin structure.

### Loss of MRC results in the early initiation of B-type granules

**Figure 7.**
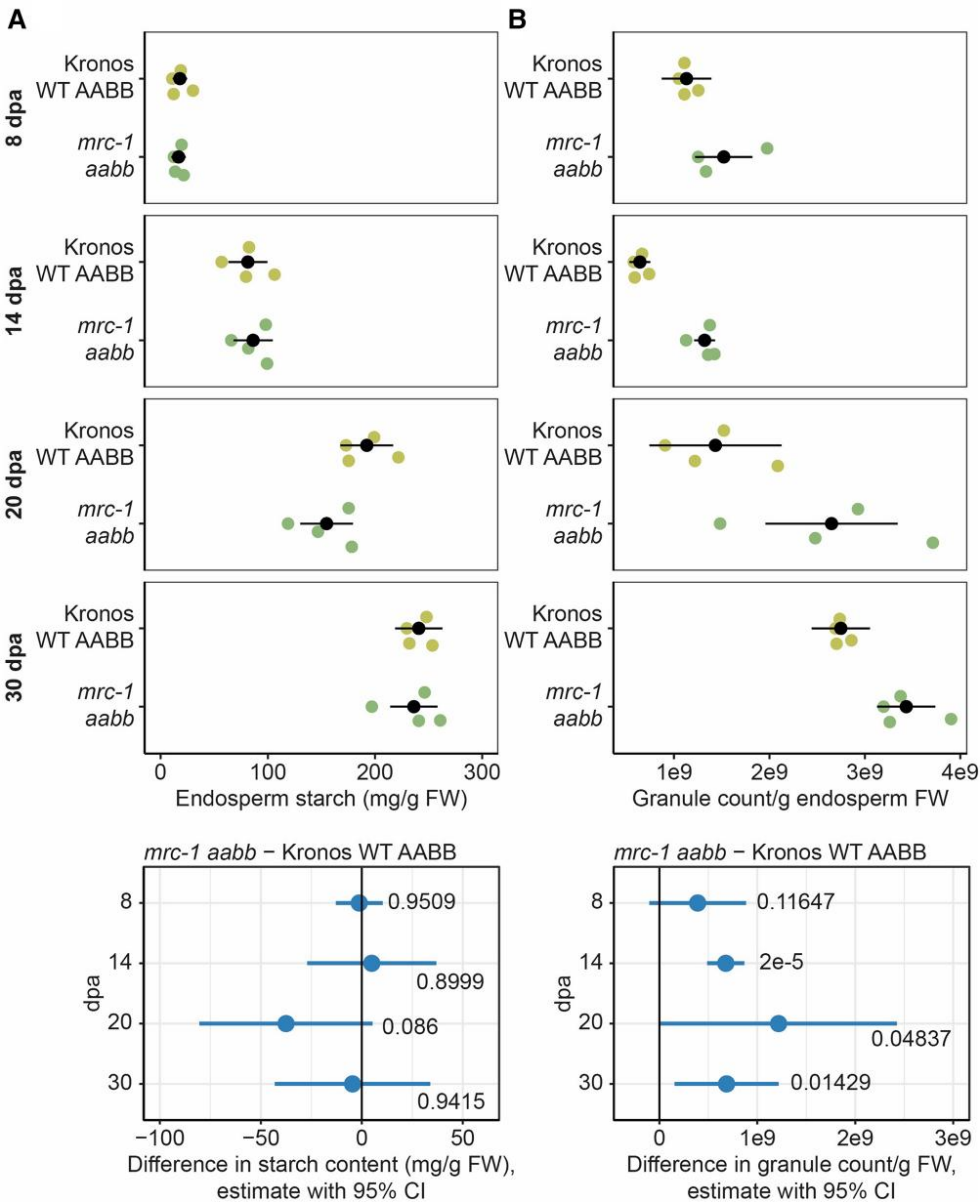
In developing endosperm, granule number increases in *mrc-1* compared to wild type (WT) despite having similar starch content. The endosperm was dissected from developing grains of WT and *mrc-1*, harvested at 8, 14, 20, and 30 d post-anthesis (dpa), with *n* = 3 to 4 individual plants for each genotype per time point, indicated by colored dots in the upper panels, with the mean ± 95% confidence interval (CI) in black dots and whiskers. **A)** Starch content of the endosperm. Values are expressed relative to the fresh weight of the dissected endosperm. **B)** Starch granule number in the endosperm. Starch was purified from dissected endosperm and the number of granules was determined using a Coulter counter in volumetric mode. Values are expressed relative to the fresh weight of the dissected endosperm. For **A** and **B)**, individual linear models were fitted to the data of each time point, with a one-way ANOVA and Tukey post hoc test to compare the means of WT and *mrc-1*. Panels on the bottom summarize the differences in means from these linear models, indicated by a dot, with whiskers showing the 95% CI of this difference, with the corresponding *P*-value.

To understand how MRC affects the size distribution of endosperm starch granules, and its specific effects on A-type or B-type granules, we investigated granule initiation during grain development in the *mrc* mutant with the strongest phenotype, *mrc-1*. We measured the total starch content and number of starch granules in dissected endosperms of developing grains harvested 8, 14, 20, and 30 days post-anthesis (dpa). The total starch content of the endosperm increased between each time point, and there was no significant difference between the mutant and the WT at any time point (Fig. 7A). At the 8 dpa time point, the mutant and the WT contained similar numbers of starch granules. Interestingly, for the two subsequent time points (14 dpa and 20 dpa), the mutant endosperms contained almost twice as many starch granules as the WT, despite similar starch contents (Fig. 7B). The largest increase in granule number during grain filling was observed between the 20 and 30 dpa time points in the WT, and between the 14 and 20 dpa time points in the mutant. At the 30 dpa time point, the difference in granule number between the mutant and WT decreased. We also noted that in both the WT and mutant, the number of starch granules decreased between the 8 and 14 dpa time points. The reason for this is unknown, but it has also been observed in *Aegilops* species—which are close relatives of wheat (Howard et al. 2011).

**Figure 8.**
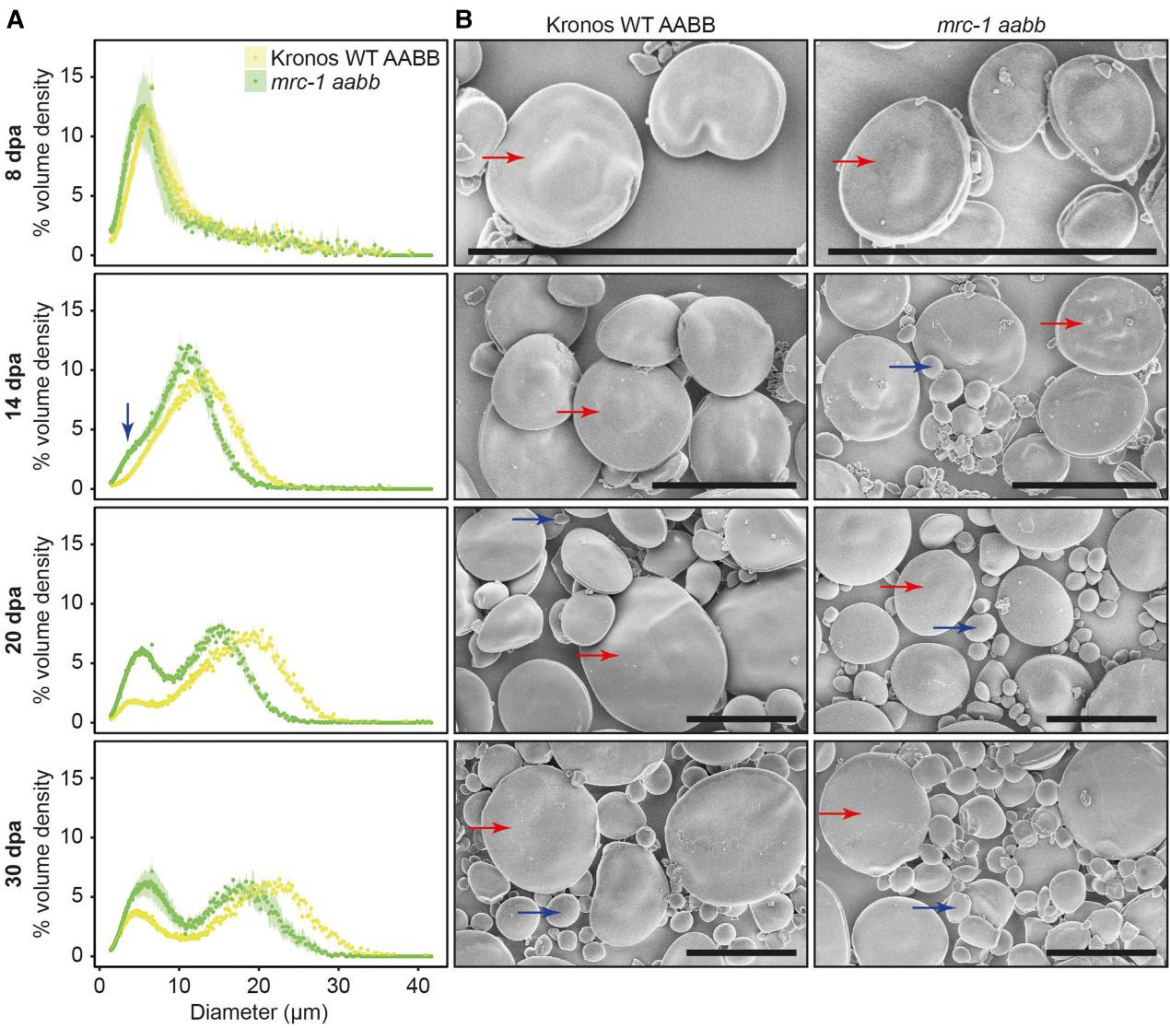
The *mrc-1* mutant initiates B-type granules earlier in grain development than in the wild type (WT). The endosperm was dissected from developing grains of WT and *mrc-1* harvested at 8, 14, 20, and 30 d post-anthesis (dpa). **A)** Coulter counter traces (measured in volumetric mode) with evenly binned *x* axes show the starch granule size distribution of endosperm starch. Distributions are the average of *n* = 4 measurements, each carried out on grains harvested from a different plant (3 grains per measurement). Data points are mean values from these 4 measurements, with the standard error of the mean shown as a shaded ribbon. The B-type granule peak in the *mrc-1* mutant at 14 dpa is indicated with a blue arrow. **B)** Endosperm starch granules were observed using scanning electron microscopy (SEM). Bars = 20 *µ*m. Examples of A-type granules and B-type granules are marked with red and blue arrows, respectively.

In WT endosperm, there was a unimodal distribution of starch granule sizes at the 8 and 14 dpa time points, and only A-type granules with their characteristic flattened morphology were observed using SEM (Fig. 8). The A-type granules grew substantially in size between the two time points, seen as a shift in the granule size distribution peak. B-type granules only became prominent at 20 dpa. In the *mrc-1* mutant, A-type granules were initially the same size as those of WT (at 8 dpa), but subsequently grew more slowly than wild-type granules. By contrast, B-type granules were already present at 14 dpa in the *mrc-1* mutant, (seen as a distinct shoulder that appeared in the granule size distribution), considerably earlier than in the WT. Taken together, these data suggest that the larger number of granules observed between 14 and 20 dpa in the *mrc-1* endosperm compared to the WT (observed in Fig. 7B) is due to the early initiation of B-type granules in the mutant.

**Figure 9.**
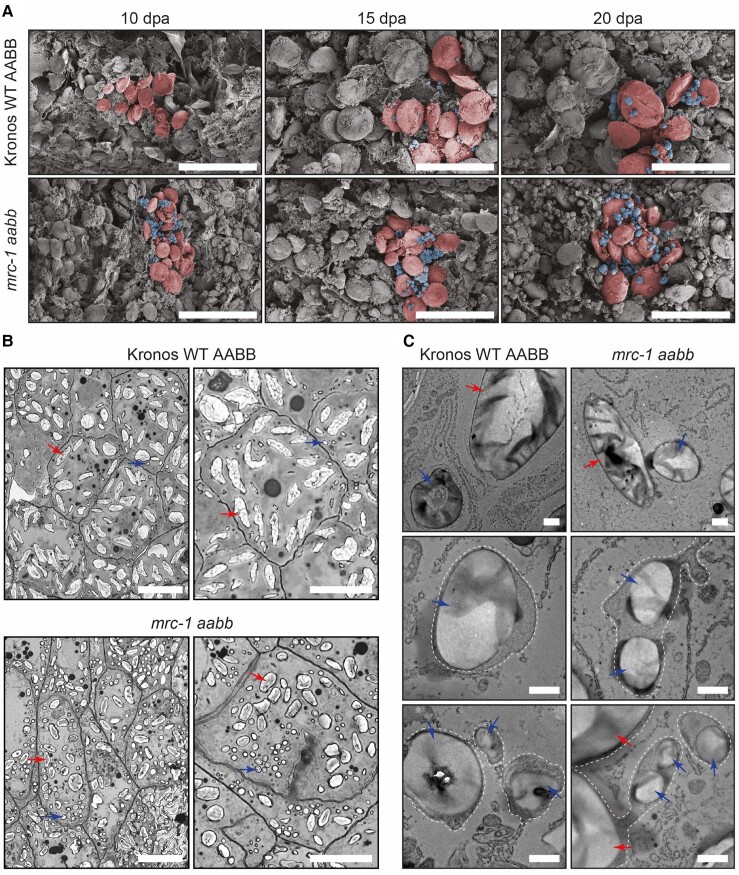
The early initiating B-type granules in *mrc-1* occur at least partially in stromules. **A)** Scanning electron micrographs of developing endosperm tissue subjected to critical point drying. Grains were harvested from wild type (WT) and *mrc-1* at 10, 15, and 20 d post-anthesis (dpa). A representative region of each panel has been pseudo-colored, with A-type granules in red shading and B-type granules in blue shading. Bars = 50 *µ*m. **B)** Light micrographs of endosperm sections. Semi-thin sections of embedded developing grains (15 dpa) were stained with toluidine blue as a negative stain for starch granules. Examples of A-type granules and B-type granules are marked with red and blue arrows, respectively. Bars = 50 *µ*m. **C)** Endosperm sections observed using transmission electron microscopy, using the same samples as in **B**. The periphery of amyloplast membranes, where visible, is indicated with a dotted white line. Bars = 1 *µ*m.

Given the strong effect of *mrc-1* on the timing of B-type granule initiation, we also explored whether the mutation affected the location of B-type granules. B-type granules typically initiate in close proximity to each other—appearing as “clusters” in between the A-type granules—and at least some B-type granules form in amyloplast stromules (Parker 1985; Langeveld et al. 2000; Esch et al. 2023). To examine B-type granules in developing grains, as opposed to the mature grains analyzed in Fig. 6, we harvested grains during their development (10, 15, and 20 dpa), subjected them to critical point drying, and imaged the cut face of sections through the endosperm using SEM. Consistent with the findings from the purified starch granules (Fig. 8), B-type granules were already present at 10 dpa in the mutant, whereas they only became prominent after 20 dpa in the WT (Fig. 9A). The B-type granules occurred in clusters in the mutant that resembled those of the WT. We also examined sections of developing grains using light and electron microscopy. For light microscopy, sections were stained with toluidine blue (a negative stain for starch). At 15 dpa, most starch granules in the wild-type endosperm were flattened A-type granules, and very few B-type granules were visible (Fig. 9B). However, in the endosperm of the *mrc-1* mutant, many clusters of B-type granules were present. Using transmission electron microscopy (TEM), we investigated the location of these granules relative to the amyloplast membranes. B-type granules were enclosed within the membranes of amyloplasts, often in elongated structures that resembled stromules. Overall, we did not observe anything unusual about the location of B-type granules in the *mrc-1* mutant.

In conclusion, MRC is required for the temporal control of B-type granule initiation during wheat grain development. It is expressed during early grain development, and its loss leads to the early initiation of B-type granules. We therefore propose that MRC acts as a repressor of B-type granule formation in the wheat endosperm during early grain development.

### Wheat MRC interacts with BGC1 in yeast-2-hybrid experiments

MRC is predicted to consist of coiled-coils, and likely acts in granule initiation through interaction with other proteins, such as BGC1. In Arabidopsis, MRC was pulled down in an immunoprecipitation with PTST2 (BGC1) (Seung et al. 2017). Since BGC1 has a crucial role in promoting B-type granule initiation in wheat, we used yeast 2-hybrid (Y2H) to investigate whether the wheat MRC and BGC1 proteins can also interact. Indeed, the Y2H showed a positive signal for the interaction with MRC and BGC1, suggesting that the proteins can participate in a direct pairwise interaction (Supplementary Fig. S4). However, the L289F mutation present in *mrc-3* did not disrupt the interaction of MRC with BGC1 in Y2H, so the loss of function of MRC in *mrc-3* may not be caused by the loss of MRC–BGC1 interaction per se. The phenotypic effects of *mrc-3* are also less severe than *mrc-1*, so perhaps there are additional effects due to the loss of MRC as a BGC1 interaction partner in *mrc-1*. Despite our best efforts, wheat MRC was recalcitrant to heterologous expression in *Escherichia coli* and in *Nicotiana benthamiana* leaves, which prevented us from verifying the interaction observed in Y2H with additional techniques. Thus, the Y2H suggests that the previously observed BGC1–MRC interaction may also occur in the wheat proteins, and future work should focus on confirming this interaction directly in the wheat endosperm.

## Discussion

### A role for MRC in repressing B-type granule initiation during endosperm starch synthesis

Starch granule initiation remains the most enigmatic part of the starch synthesis process, where we understand little about how the diverse numbers and morphologies of starch granules are determined in our most important crops (Seung and Smith 2019; Abt and Zeeman 2020; Chen et al. 2021). Here, we shed light on the temporal regulation of granule initiation in wheat endosperm, by demonstrating the unique role of MRC in the repression of B-type granule initiation during early grain development.

The expression pattern of MRC during grain development is consistent with the change in onset of B-type granule initiation observed in the mutant. MRC is expressed in the endosperm between 6 and 10 dpa but decreases rapidly in expression between 10 and 15 dpa, remaining low after 15 dpa (Fig. 2). This decline in expression coincides with when B-type granules start to form in the WT (between 14 and 20 dpa; Figs. 7 to 9). In the *mrc-1* mutant, B-type granules were initiated earlier than in the WT, already at 10 dpa (Figs. 8 and 9), which could be due to the loss of B-type granule repression by MRC in early grain development. There was a strong increase in starch granule number in the *mrc-1* mutant compared to WT at 14 and 20 dpa. Considering that this gap in number narrowed at 30 dpa (Fig. 7), and that there were only small differences in granule number in the mature grains (Fig. 4B), it seems that loss of MRC results in an initial boost in granule number due to the early B-granule initiation, and that this plateaus to a similar number as the WT in mature grains.

**Figure 10.**
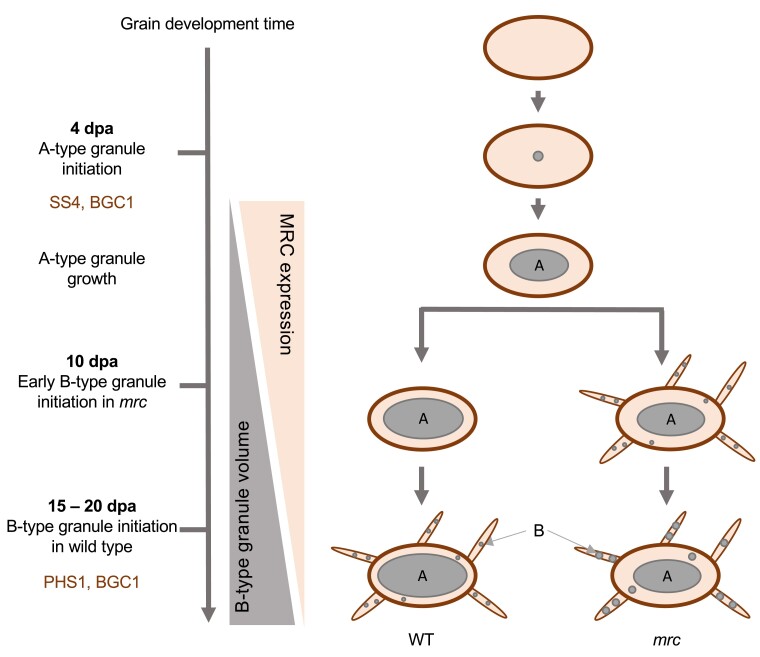
Model of MRC function in B-type granule initiation in developing wheat endosperm. MRC is required for the control of the timing of B-type granule initiation during early grain development. In wild type (WT), A-type granules initiate around 4 d post-anthesis (dpa), through action of SS4 and BGC1, and B-type granules initiate around 15 to 20 dpa, through action of PHS1 and BGC1. We propose that the expression of MRC during early endosperm development (∼10 dpa) prevents the B-type granule formation, and B-type granule volume increases as MRC expression decreases after 10 dpa. This process is disrupted in mutants lacking a functional MRC protein, and granules initiate already from 10 dpa. The early initiation of B-type granules provides them with more time and substrate to grow, leading to higher volume of B-type granules in the mutant at grain maturity compared to the wild type, mostly contributed by an increase in B-type granule size, and concomitantly reduced growth in A-type granule size.

We propose that the early initiation of B-type granules results in drastically altered endosperm starch granule size distributions in the mutants (Fig. 10). The early appearance of B-type granules in the *mrc-1* mutant could present competition with A-type granules for substrates for granule growth (i.e. ADP-glucose) from an earlier stage of grain development than in the WT, resulting in larger B-type granules and smaller A-type granules in the mature mutant grains (Fig. 5). At 8 dpa, before the appearance of B-type granules, the size distribution curves of A-type granules were almost identical between mutant and WT (Fig. 8), and it was only at the later stages of grain development following B-type granule initiation that the A-type granules became smaller in the mutant compared to WT. The lack of difference in A-type granule number and size at 8 dpa suggests that the *mrc-1* mutation did not affect A-type granule initiation. Both *phs1* and *mrc* mutants have larger B-type granules. In *phs1*, this is likely caused by the drastic decrease in B-type granule number, creating less competition for substrates among the B-type granules (Kamble et al. 2023). However, in *mrc* mutants, the crucial difference is in the earlier timing of B-type granule initiation, which creates competition for substrates with developing A-type granules.

The higher relative B-type granule volume could be caused by a combination of smaller A-type granules and larger B-type granules. It is difficult to make direct conclusions about the effect of MRC on B-type granule number specifically, considering our definition of B-type granules comes from curve fitting volume/size distribution graphs from the Coulter counter. However, there were no big changes in the proportion of small granules in the mutants (which are mostly B-type granules) (Fig. 4C), suggesting that the early initiation of B-type granules does not necessarily result in a measurable increase in B-type granule number. Small differences in B-type granule number and size may be difficult to detect as the B-type granules already make up a much larger proportion of the total granule number in WT. For *mrc-3*, a higher B-type granule volume percentage was observed (Fig. 5B) without a measurable increase in B-type granule size or in total granule number (Fig. 4B).

Therefore, despite all *mrc* mutants having an increased proportion of B-type granule volume, they differed in severity (Fig. 5). The strongest effect on B-type granule percentage was seen in *mrc-1*, and since the phenotypes of the *mrc-1* backcrossed and non-backcrossed lines were similar, the severity of this line is not due to background mutations. The *mrc-1* line may have the strongest phenotype because the premature stop codon occurs earlier in the coding sequence than in *mrc-2*, and it is possible that the truncated protein in *mrc-2* is partially functional. The *mrc-3* mutant had the weakest phenotype as it is a missense mutation, and suggests that the Leu289Phe mutation greatly inhibits, but does not eliminate MRC function.

The *mrc-1* wild-type segregant likely has some background mutations, as several of its phenotypes differed from the WT. The presence of background mutations is not unusual in these EMS-mutagenized wheat TILLING lines (Uauy et al. 2017), and importantly, the effects observed in the wild-type segregant were minor compared to the effects observed in the mutants.

### Exploring the biochemical basis of MRC function in B-type granule initiation

Since MRC is a long coiled-coil protein with no known enzymatic domains, it is possible that it can exert specific effects on granule initiation by interacting with different interaction partners. Recent work has begun to uncover the distinct roles of Arabidopsis starch granule initiation orthologs in wheat, with BGC1 and MRC both having roles in B-type granule initiation. However, a B-type granule suppressing mechanism during early grain development, like we report here for MRC, has not previously been described. It is tempting to speculate that MRC may influence the timing of B-type granule initiation by interacting with BGC1. Interactions between MRC and PTST2 (BGC1) have been shown for the Arabidopsis proteins (Seung et al. 2017). Our Y2H experiment suggested that wheat MRC and BGC1 can also interact directly (Supplementary Fig. S4). However, we were not able to confirm this interaction using additional methods due to technical difficulties in expressing MRC in heterologous systems. The potential BGC1–MRC interaction will need to be further investigated in a physiological context, preferably using antibodies to probe the interaction directly in endosperm extracts. The mutant phenotypes in this study suggest that MRC might have opposing roles to BGC1 and PHS1 in the wheat endosperm, despite the similar initiation promoting roles of MRC and PTST2 seen in leaves of Arabidopsis (Seung et al. 2017, 2018) and wheat (Watson-Lazowski et al. 2022). The repressive role of MRC in B-type granule initiation in wheat endosperm could be facilitated by it binding and inhibiting the action of BGC1, or the MRC–BGC1 interaction could specifically promote A-type granule initiation rather than B-type granule initiation. Future studies will have to uncover whether this is due to a difference in the context of the biochemical interaction between MRC and PTST2/BGC1 in Arabidopsis leaves and wheat endosperm.

Future studies on the dynamics of biochemical interactions through time will provide a clearer view on the action of MRC. A previous pull-down of BGC1 in wheat endosperm at 18 dpa did not capture MRC (Kamble et al. 2023), which is to be expected as the expression of MRC is low at that developmental time point. It would be interesting to see if BGC1-interacting proteins are different during early endosperm development. PHS1 expression also peaks during early endosperm development (10 dpa), but remains high throughout further grain development, and the relationship between PHS1 and MRC remains to be explored.

The striking effect of the L289F mutation in the *mrc-3* mutant on endosperm starch may provide an important clue in functional studies, since *mrc-3* had an increased B-type granule percentage in the endosperm, with only a single point mutation. Y2H experiments demonstrate that the L289F mutation does not influence the ability of MRC and BGC1 to interact, but the L289F mutation could affect protein stability, conformation, localization or other interactions, influencing the in vivo dynamics of granule initiation proteins. Understanding these effects might also help dissect the differences in MRC function in Arabidopsis leaves versus wheat endosperm. The Arabidopsis granule initiation proteins localize to distinct puncta in the chloroplast, but we do not yet know much about the localization of these wheat orthologs in the chloroplast or amyloplast.

### MRC as a gene target for biotechnological modification of starch granule size

Overall, our discovery that MRC plays an important role in the timing of B-type granule initiation highlights the complex regulation of the process during grain development. This raises a pertinent question as to whether there is an adaptive advantage of having A- and B-type granules in the endosperm of the Triticeae. Indeed, there could be a specific function for B-type granules, but more work is needed to determine this function. For example, we now have wheat mutants with varying B-type granule contents, including *mrc*, *bgc1*, and *phs1* mutants (Chia et al. 2020; Kamble et al. 2023), which provides the ideal genetic material to test how B-type granules affect seed fitness and germination rates.

Despite not knowing the adaptive advantage for MRC and B-type granules in the plant, there is already great potential for using MRC to manipulate starch properties for biotechnological purposes. There is great industrial interest in manipulating starch granule size in crop species, as granule size affects the physico-chemical properties of starch and digestibility (Jobling 2004; Lindeboom et al. 2004; Chen et al. 2021; Li et al. 2023). Our results establish MRC as a promising gene target for modifying starch granule size distribution in wheat, specifically to achieve smaller starch granules and a narrower granule size distribution range than conventional cultivars. Small granules are more efficiently digested in vitro than large granules, due to their larger surface area to volume ratio (Dhital et al. 2010). Possible uses for wheat *mrc* starch within the food industry include pasta making, where more B-type granules positively affect pasta quality due to their higher rate of water absorption (Soh et al. 2006). Wheat *mrc* starch may also be useful in industrial applications like papermaking and biodegradable plastics, where small granules are desirable (Lindeboom et al. 2004). Functional tests can be directly performed on our material to provide proof of concept that the altered granule size distribution in the *mrc* mutants improves grain/starch quality.

MRC is a promising target because different granule size can be achieved without accompanying effects on overall plant growth (Fig. 3), on grain weight and total starch content (Fig. 4), on starch granule shape (Fig. 6), or on amylopectin structure and amylose content (Supplementary Fig. S2). It is also ideal that the B-genome homeolog has become a pseudogene (Fig. 1), meaning that only 1 or 2 homeologs need to be mutated in durum and bread wheat, respectively, to achieve an effect on granule size. Further, we have also demonstrated that different mutations in MRC can be used to fine-tune the volume of B-type granules in endosperm starch, such as those in *mrc-2* and *mrc-3* to achieve moderate increases, and *mrc-1* to achieve larger increases. We are currently investigating whether overexpression of MRC can be used to reduce the volume of B-type granules.

Since the repression of B-type granule initiation is likely to be a role specific to Triticeae species carrying a bimodal size distribution of endosperm starch, it remains to be determined what the role of MRC is in cereal species that do not have a bimodal distribution of starch granules, such as those that have compound granules (e.g. in rice). Also, oats have a bimodal distribution of starch granules, with large compound granules and smaller simple granules. However, in oat, the smaller granules initiate at the same time as the larger compound granules (Saccomanno et al. 2017), and it would therefore be interesting to determine if differences in MRC function play a role in timing the initiation of the small granules during oat endosperm development. Exploring the role of MRC in multiple crop species would therefore not only reveal the molecular differences that result in distinct spatiotemporal patterns of granule initiation between species, but could also increase its biotechnological potential.

## Materials and methods

### TaMRC bioinformatic analysis

To characterize *TtMRC-B1* in tetraploid wheats, we aligned the whole genome-sequencing reads of *T. dicoccoides* (*n* = 10) and *T. turgidum* ssp. *durum* (*n* = 10) from Zhou et al. (2020) against the “tetraploid” version of the Ref-Seqv1.0 Chinese Spring assembly (Appels et al. 2018). We used HiSat2-v-2.1.0 (Kim et al. 2019) with the default settings and visualized the read alignments on the genetic signatures of retrotransposon insertion on *MRC-B1* (Fig. 1B) using Integrated Genomics Viewer (Robinson et al. 2011). Phylogenetic analyses were performed as described in Seung et al. (2018).

### Plant materials and growth

EMS mutants of tetraploid wheat (*T. turgidum* cv. ‘Kronos’) carrying mutations in *TtMRC-A1* and the chromosome 6B pseudogene were identified from the wheat in silico TILLING database (http://www.wheat-tilling.com; Krasileva et al. 2017) and obtained from the John Innes Centre Germplasm Resource Unit. The selected mutants for *TtMRC-A1* were Kronos3272 (K3272), Kronos598 (K598), and Kronos4681 (K4681); while Kronos4305 (K4305) and Kronos3078 (K3078) were selected for the 6B pseudogene. From these mutants, we generated 3 different sets of lines. The *mrc-1* lines descend from a cross between K3272 and K3078, while the *mrc-2* lines descend from a cross between K4681 and K4305. For both crosses, *aa*BB, AA*bb*, and *aabb* genotypes were obtained in the F2 generation. The *mrc-3* lines are the original K598 mutants. The KASP markers used to genotype the mutations are provided in Supplementary Table S4.

Plants were grown in soil in a controlled environment room with fluorescent lamps supplemented with LED panels. The chambers were set to provide a 16-h light at 300 *µ*mol photons m^−2^ s^−1^ and 20 °C, and 8-h dark at 16 °C, with relative humidity of 60%. Grains from the first 3 tillers were harvested from mature, dry spikes (approximately 4 mo after sowing).

### Grain morphometrics

The number of grains harvested per plant, plus grain area, and TGW were quantified using the MARViN seed analyzer (Marvitech GmbH, Wittenburg). Multiple grains from each plant (15 to 88 individual grains per plant) were measured for grain area to calculate the average value for each plant, and these values were used in the plots of Fig. 3F and the analysis of Supplementary Table S3D.

### Starch purification from mature grains or developing endosperm

Starch was purified from mature grains using 3 to 6 grains per extraction. Dry grains were soaked overnight at 4 °C in 5 mL of sterile water. The softened grains were homogenized in 10 mL sterile water using a mortar and pestle, and the homogenate was filtered through a 100 *µ*m mesh. The starch was pelleted by centrifugation at 3,000 × *g* for 5 min, and resuspended in 2 mL of water. The resuspended starch was loaded on top of a 5 mL 90% (v/v) Percoll (Sigma) cushion buffered with 50 mm Tris-HCl, pH 8, and was spun at 2,500 × *g* for 15 min. We verified that no intact granules were left in the Percoll interface after the spin. The starch pellet was washed twice with wash buffer (50 mm Tris-HCl, pH 6.8; 10 mm EDTA; 4% (w/v) SDS; and 10 mm DTT), then 3 times with water, followed by a final wash in absolute ethanol. The starch was then air-dried overnight.

For starch extraction from developing endosperm, developing grains were harvested at the indicated time points and were snap frozen in liquid nitrogen and stored at −80 °C until analysis. Each grain was thawed just prior to extraction and the endosperm was carefully dissected and placed into a chilled tube and weighed. The tissue was then homogenized in sterile water with a pestle, then filtered through a 60 *µ*m mesh. The pellet was washed 3 times in 90% (v/v) Percoll (Sigma) buffered with 50 mm Tris-HCl, pH 8, then 3 times with wash buffer (as above), followed by 3 times with water.

### Coulter counter analysis of starch granule size and number

For profiles of granule size distribution, purified starch was suspended in Isoton II diluent (Beckman Coulter) and analyzed with a Multisizer 4e Coulter counter fitted with a 70 *µ*m aperture (Beckman Coulter). Granules were counted either in volumetric mode (all data in main figures), measuring 1 mL from a total 100 mL volume preparation containing 20 *µ*L of purified starch or set to count at least 100,000 granules (Supplementary Fig. S3). For calculations of granule counts in volumetric mode, the number of granules per mg grain weight was back calculated to the total starting grain weight. For determination of percentage of small granules relative to total granule number, the number of granules that were <10 *μ*m were selected and divided by the total number of granules. The granules were sized, with the Coulter counter collecting the data using logarithmic bins for the granule diameter (standard settings).

For each plant, to calculate the mean A- and B-type granule size, as well as relative B-type granule volume, we fitted a mixed bimodal Gaussian curve to the distribution using R (https://github.com/JIC-CSB/coulter_counter_fitting). As the data collection on the Coulter counter is set to logarithmic bins on the *x* axis, for these calculations and for the traces in Figs. 5A and 8A and Supplementary Fig. S3, we transformed the *x* axis to even bins, by changing the *y* axis to volume percentage density (volume percentage for each bin divided by bin width). For each of the extracted phenotypes of mean A- and B-type granule size and relative B-type granule volume, we fitted individual linear models and performed a one-way ANOVA and Tukey post hoc tests for pairwise comparisons of the genotypes, using the lm() and emmeans() functions in R, from the “stats” and “emmeans” packages. Nine individual plants per genotype were used for this experiment.

### Light and electron microscopy

For light microscopy of endosperm sections from mature grains, thin sections (1 *µ*m thick) of mature grains were made using a microtome fitted with a glass knife. Sections were mounted onto a glass slide and stained with 3% (v/v) Lugol's iodine solution (Sigma) prior to imaging on a DM6000 (Leica) or AxioObserver Z1 (Zeiss) microscope.

For light/electron microscopy of developing endosperm tissue, developing grains (15 dpa) were harvested into 4% (w/v) paraformaldehyde, 2.5% (w/v) glutaraldehyde in 0.05 m sodium cacodylate, pH 7.4. The segments were post-fixed with osmium, then dehydrated in an ascending ethanol series, and embedded in LR white resin using an EM TP embedding machine (Leica). For light microscopy, semi-thin sections (0.5 *µ*m thick) were produced from the embedded samples using a glass knife and were dried onto PTFE-coated slides. The sections were stained with toluidine blue and imaged as described above.

For TEM, ultra-thin sections (approximately 90 nm) were produced from the embedded grains by sectioning with a diamond knife using a Leica UC7 ultramicrotome (Leica, Milton Keynes). The sections were picked up on 200 mesh copper grids which had been formvar and carbon coated, then stained with 2% (w/v) uranyl acetate for 1 h and 1% (w/v) lead citrate for 1 min, washed in distilled water and air-dried. The grids were viewed in a FEI Talos 200C transmission electron microscope (FEI UK Ltd, Cambridge, UK) at 200 kV and imaged using a Gatan OneView 4 K × 4 K digital camera (Gatan, Cambridge, UK) to record DM4 files.

For SEM: For imaging starch granules, a drop of purified starch suspended in water (5 mg/mL) was air-dried onto a glass coverslip attached onto an SEM stub. For imaging sections through developing endosperm, harvested grains were fixed in 2.5% (w/v) glutaraldehyde in 0.05 m sodium cacodylate, pH 7.4. The fixative was removed by washing with 0.05 m sodium cacodylate, pH 7.4, after which the grains were dehydrated in an ascending ethanol series, and then subjected to critical point drying in a CPD300 instrument (Leica) according to the manufacturer's instructions. Thick transverse sections were produced from the dried grains and were glued onto SEM stubs. All stubs were sputter coated with gold and observed using either a Supra 55 VPFEG (Zeiss) or Nova NanoSEM 450 (FEI) SEM instrument.

### Quantification of starch content in endosperm

Mature grains (5 to 6 grains) were soaked overnight at 4 °C in 5 mL of sterile water and were homogenized using a mortar and pestle. Developing endosperm tissue was extracted in 1 mL of sterile water with the pestle. Insoluble material in an aliquot of the homogenate was collected by centrifugation at 5,000 × *g* for 5 min, then washed once in 0.7 m perchloric acid, once in sterile water, then 3 times in 80% (v/v) ethanol. The pellet was then resuspended in water. Starch in the pellet was gelatinized by heating at 95 °C for 15 min, then digested using α-amylase (Megazyme) and amyloglucosidase (Roche). The glucose released was measured using the hexokinase/glucose-6-phosphate dehydrogenase method (Roche). Starch content (in glucose equivalents) was calculated relative to the original dry weight of the analyzed grains.

### Analysis of amylopectin structure and amylose content

Amylopectin structure and amylose content were analyzed using purified starch. Amylopectin structure in terms of chain length distribution was quantified using High Performance Anion Exchange Chromatography with Pulsed Amperometric Detection (HPAEC-PAD) (Blennow et al. 1998). For amylose content, granules were dispersed in DMSO and quantified using an iodine-binding method (Warren et al. 2016).

### Y2H

For the Y2H assay, the full-length coding sequence of *Ta*MRC was synthesized as a gBlocks gene fragment (IDT DNA) (codon optimized for *A. thaliana* to reduce sequence complexity—sequence in Supplementary File 2), flanked with attB1 and attB2 Gateway recombination sites. This was recombined into the pDONR221 vector using BP clonase II (Thermo Fisher, Loughborough, UK). The coding sequence of *Ta*MRC and *Ta*BGC1 (using the BGC1:pDONR221 vector—Hawkins et al. 2021) were then recombined using LR clonase (Thermo Fisher) into the Gateway-compatible vectors pGBKT7 and pGADT7 (Takara Bio, London, UK). The *Ta*MRC L289F construct was generated using the Q5 site-directed mutagenesis kit (New England Biolabs, Hitchin, UK) and the primers 5′-CGAGCAAGAATTTGAAAAACAGAG-3′ and 5′-GCGACCTTTAACTTCTCC-3′.

The Gal4-based Y2H assay (Fields and Song 1989) was performed using the yeast (*Saccharomyces cerevisiae*) strain AH109. Approx. 200 ng of each plasmid was added in the relevant combinations to competent AH109 cells (100 *μ*L) in sterile 100 mm lithium acetate, and 281 *μ*L of transformation mixture (240 *μ*L 50% (w/v) PEG, 36 *μ*L 1 m LiOAc, 5 *μ*L carrier DNA [Takara Bio]) was added to each combination. The mixture was vortexed for 1 min, incubated at 30 °C for 30 min, then incubated at 42 °C for 30 min, and centrifuged for 5 min at 700 × *g*. The pellet was resuspended in 100 *μ*L of 0.9% (w/v) NaCl and each transformation was plated on double dropout (yeast medium without leucine and tryptophan (-LW), Y0750, Merck) yeast selection plates with SD + agar (6.9 g/L yeast nitrogen base without amino acids (Formedium), 20 g/L glucose, 20 g/L agar), and incubated at 30 °C for 2 to 3 d. A clump of yeast cells from successful transformations was used to inoculate 5 mL SD + -LW cultures, and the cultures were incubated at 28 to 30 °C at 200 rpm overnight. A total of 2 mL of each culture was centrifuged at 10,621 × *g* for 5 min, and the pellets were resuspended in 1 mL sterile water. Serial dilutions of these cultures were made: no dilution, 1 in 10, 1 in 100, and 1 in 500. A total of 10 *μ*L of each dilution for each transformation was spotted on both a -LW SD + agar plate and a quadruple dropout SD + agar plate (-LWHA: medium without leucine, tryptophan, histidine, adenine, Y2021, Merck). These plates were left to dry in a laminar flow cabinet and were incubated at 30 °C for 3 d. For the images used, the plates had been kept at room temperature for a few extra days.

### Statistics

All statistical analyses were done in R version 4. Overall, we used the emmeans() function from the “emmeans” package throughout for pairwise comparisons. For most experiments, we used linear models using the lm() function from the “stats” package and did one-way ANOVAs with Tukey post hoc tests. Details for individual experiments where we used other models (Poisson regression) are described for the relevant sections. Figure legends and Supplementary Table S3 also describe details of statistics for individual experiments.

### Accession numbers

The accession numbers corresponding to the genes investigated in this study are*: Tt*MRC-A1 (TRITD6Av1G081580), *Ta*MRC-A1 (TraesCS6A02G180500), and *Ta*MRC-D1 (TraesCS6D02G164600).

## Supplementary Material

kiae429_Supplementary_Data

## Data Availability

The data underlying this article are available in the article and in its online supplementary material.

## Supplementary data

The following materials are available in the online version of this article.

**Supplementary File 1.**  *Aegilops speltoides* MRC gene model.

**Supplementary File 2.** Codon optimized sequence for TaMRC.

**Supplementary Figure S1.** Phylogenetic analysis of wheat MRC sequences.

**Supplementary Figure S2.** Chain length distribution and amylose content of the *mrc* mutants.

**Supplementary Figure S3.** The 6B pseudogene does not contribute to granule size distribution in wheat endosperm starch.

**Supplementary Figure S4.** Direct interaction between *Ta*MRC and *Ta*BGC1 in yeast 2-hybrid (Y2H).

**Supplementary Table S1.** Amino acid identity and similarity values between Arabidopsis and wheat MRC.

**Supplementary Table S2.** Reads mapped to retrotransposon insertion genetic signatures on *MRC-B1*.

**Supplementary Table S3.** Pairwise comparisons of wheat growth phenotypes.

**Supplementary Table S4.** KASP markers for genotyping the wheat mutants.

## Dive Curated Terms

The following phenotypic, genotypic, and functional terms are of significance to the work described in this paper:

PHS1 Gramene: At3g29320
PHS1 Araport: At3g29320

starch CHEBI: CHEBI:28017

endosperm AmiGo: PO:0009089

starch content Planteome: TO:0000696

amylopectin CHEBI: CHEBI:28057

TtMRC-A1 Gramene: TRITD6Av1G081580
TtMRC-A1 Araport: TRITD6Av1G081580

TaMRC-D1 Gramene: TraesCS6D02G164600
TaMRC-D1 Araport: TraesCS6D02G164600

TaMRC-A1 Gramene: TraesCS6A02G180500
TaMRC-A1 Araport: TraesCS6A02G180500

## References

[kiae429-B1] Abt MR, Zeeman SC. Evolutionary innovations in starch metabolism. Curr Opin Plant Biol. 2020:55:109–117. 10.1016/j.pbi.2020.03.001 32428846

[kiae429-B2] Appels R, Eversole K, Stein N, Feuillet C, Keller B, Rogers J, Pozniak CJ, Choulet F, Distelfeld A, Poland J, et al Shifting the limits in wheat research and breeding using a fully annotated reference genome. Science. 2018:361(6403):eaar7191. 10.1126/science.aar7191 30115783

[kiae429-B3] Bechtel DB, Zayas I, Kaleikau L, Pomeranz Y. Size-distribution of wheat starch granules during endosperm development. Cereal Chem. 1990:67:59–63.

[kiae429-B4] Blennow A, Bay-Smidt AM, Wischmann B, Olsen CE, Møller BL. The degree of starch phosphorylation is related to the chain length distribution of the neutral and the phosphorylated chains of amylopectin. Carbohydr Res. 1998:307(1–2):45–54. 10.1016/S0008-6215(98)00015-9

[kiae429-B5] Chen J, Hawkins E, Seung D. Towards targeted starch modification in plants. Curr Opin Plant Biol. 2021:60:102013. 10.1016/j.pbi.2021.102013 33677239

[kiae429-B6] Chen J, Watson-Lazowski A, Kamble NU, Vickers M, Seung D. Gene expression profile of the developing endosperm in durum wheat provides insight into starch biosynthesis. BMC Plant Biol. 2023:23(1):363. 10.1186/s12870-023-04369-7 37460981 PMC10353290

[kiae429-B7] Chia T, Chirico M, King R, Ramirez-Gonzalez R, Saccomanno B, Seung D, Simmonds J, Trick M, Uauy C, Verhoeven T, et al A carbohydrate-binding protein, B-GRANULE CONTENT 1, influences starch granule size distribution in a dose-dependent manner in polyploid wheat. J Exp Bot. 2020:71(1):105–115. 10.1093/jxb/erz405 31633795

[kiae429-B8] Daron J, Glover N, Pingault L, Theil S, Jamilloux V, Paux E, Barbe V, Mangenot S, Alberti A, Wincker P, et al Organization and evolution of transposable elements along the bread wheat chromosome 3B. Genome Biol. 2014:15(12):546. 10.1186/s13059-014-0546-4 25476263 PMC4290129

[kiae429-B9] Dhital S, Shrestha AK, Gidley MJ. Relationship between granule size and in vitro digestibility of maize and potato starches. Carbohydr Polym. 2010:82(2):480–488. 10.1016/j.carbpol.2010.05.018

[kiae429-B10] Esch L, Ngai QY, Barclay JE, McNelly R, Hayta S, Smedley MA, Smith AM, Seung D. Increasing amyloplast size in wheat endosperm through mutation of PARC6 affects starch granule morphology. New Phytol. 2023:240(1):224–241. 10.1111/nph.19118 37424336 PMC10952435

[kiae429-B11] Fields S, Song O. A novel genetic system to detect protein–protein interactions. Nature. 1989:340(6230):245–246. 10.1038/340245a0 2547163

[kiae429-B12] Hawkins E, Chen J, Watson-Lazowski A, Ahn-Jarvis J, Barclay JE, Fahy B, Hartley M, Warren FJ, Seung D. STARCH SYNTHASE 4 is required for normal starch granule initiation in amyloplasts of wheat endosperm. New Phytol. 2021:230(6):2371–2386. 10.1111/nph.17342 33714222

[kiae429-B13] Howard T, Rejab NA, Griffiths S, Leigh F, Leverington-Waite M, Simmonds J, Uauy C, Trafford K. Identification of a major QTL controlling the content of B-type starch granules in Aegilops. J Exp Bot. 2011:62(6):2217–2228. 10.1093/jxb/erq423 21227932 PMC3060699

[kiae429-B14] Jobling S . Improving starch for food and industrial applications. Curr Opin Plant Biol. 2004:7(2):210–218. 10.1016/j.pbi.2003.12.001 15003223

[kiae429-B15] Kamble NU, Makhamadjonov F, Fahy B, Martins C, Saalbach G, Seung D. Initiation of B-type starch granules in wheat endosperm requires the plastidial α-glucan phosphorylase PHS1. Plant Cell. 2023:35(11):4091–4110. 10.1093/plcell/koad217 37595145 PMC10615211

[kiae429-B16] Kim D, Paggi JM, Park C, Bennett C, Salzberg SL. Graph-based genome alignment and genotyping with HISAT2 and HISAT-genotype. Nat Biotechnol. 2019:37(8):907–915. 10.1038/s41587-019-0201-4 31375807 PMC7605509

[kiae429-B17] Krasileva KV, Vasquez-Gross HA, Howell T, Bailey P, Paraiso F, Clissold L, Simmonds J, Ramirez-Gonzalez RH, Wang X, Borrill P, et al Uncovering hidden variation in polyploid wheat. Proc Natl Acad Sci U S A. 2017:114(6):E913–E921. 10.1073/pnas.1619268114 28096351 PMC5307431

[kiae429-B18] Langeveld SMJ, Van wijk R, Stuurman N, Kijne JW, de Pater S. B-type granule containing protrusions and interconnections between amyloplasts in developing wheat endosperm revealed by transmission electron microscopy and GFP expression. J Exp Bot. 2000:51(349):1357–1361. 10.1093/jexbot/51.349.1357 10944148

[kiae429-B19] Li LF, Zhang ZB, Wang ZH, Li N, Sha Y, Wang XF, Ding N, Li Y, Zhao J, Wu Y, et al Genome sequences of five Sitopsis species of Aegilops and the origin of polyploid wheat B subgenome. Mol Plant. 2022:15(3):488–503. 10.1016/j.molp.2021.12.019 34979290

[kiae429-B20] Li M, Daygon VD, Solah V, Dhital S. Starch granule size: does it matter? Crit Rev Food Sci Nutr. 2023:63(19):3683–3703. 10.1080/10408398.2021.1992607 34704861

[kiae429-B21] Lindeboom N, Chang PR, Tyler RT. Analytical, biochemical and physicochemical aspects of starch granule size, with emphasis on small granule starches: a review. Starch—Stärke. 2004:56(3–4):89–99. 10.1002/star.200300218

[kiae429-B22] Maccaferri M, Harris NS, Twardziok SO, Pasam RK, Gundlach H, Spannagl M, Ormanbekova D, Lux T, Prade VM, Milner SG, et al Durum wheat genome highlights past domestication signatures and future improvement targets. Nat Genet. 2019:51(5):885–895. 10.1038/s41588-019-0381-3 30962619

[kiae429-B23] Malinova I, Mahlow S, Alseekh S, Orawetz T, Fernie AR, Baumann O, Steup M, Fettke J. Double knockout mutants of Arabidopsis grown under normal conditions reveal that the plastidial phosphorylase isozyme participates in transitory starch metabolism. Plant Physiol. 2014:164(2):907–921. 10.1104/pp.113.227843 24302650 PMC3912115

[kiae429-B24] Matsushima R, Yamashita J, Kariyama S, Enomoto T, Sakamoto W. A phylogenetic re-evaluation of morphological variations of starch grains among Poaceae species. J Appl Glycosci. 2013:60(1):37–44. 10.5458/jag.jag.JAG-2012_006

[kiae429-B25] Ng PC, Henikoff S. Predicting the effects of amino acid substitutions on protein function. Annu Rev Genomics Hum Genet. 2006:7(1):61–80. 10.1146/annurev.genom.7.080505.115630 16824020

[kiae429-B26] Parker ML . The relationship between A-type and B-type starch granules in the developing endosperm of wheat. J Cereal Sci. 1985:3(4):271–278. 10.1016/S0733-5210(85)80001-1

[kiae429-B27] Peng C, Wang Y, Liu F, Ren Y, Zhou K, Lv J, Zheng M, Zhao S, Zhang L, Wang C, et al FLOURY ENDOSPERM6 encodes a CBM48 domain-containing protein involved in compound granule formation and starch synthesis in rice endosperm. Plant J. 2014:77(6):917–930. 10.1111/tpj.12444 24456533

[kiae429-B28] Robinson JT, Thorvaldsdottir H, Winckler W, Guttman M, Lander ES, Getz G, Mesirov JP. Integrative genomics viewer. Nat Biotechnol. 2011:29(1):24–26. 10.1038/nbt.1754 21221095 PMC3346182

[kiae429-B29] Roldán I, Wattebled F, Mercedes Lucas M, Delvallé D, Planchot V, Jiménez S, Pérez R, Ball S, D'Hulst C, Mérida A. The phenotype of soluble starch synthase IV defective mutants of *Arabidopsis thaliana* suggests a novel function of elongation enzymes in the control of starch granule formation. Plant J. 2007:49(3):492–504. 10.1111/j.1365-313X.2006.02968.x 17217470

[kiae429-B30] Saccomanno B, Berbezy P, Findlay K, Shoesmith J, Uauy C, Viallis B, Trafford K. Characterization of wheat lacking B-type starch granules. J Cereal Sci. 2022:104:103398. 10.1016/j.jcs.2021.103398 35340793 PMC8935375

[kiae429-B31] Saccomanno B, Chambers AH, Hayes A, Mackay I, McWilliam SC, Trafford K. Starch granule morphology in oat endosperm. J Cereal Sci. 2017:73:46–54. 10.1016/j.jcs.2016.10.011

[kiae429-B32] Saito M, Tanaka T, Sato K, Vrinten P, Nakamura T. A single nucleotide polymorphism in the “Fra” gene results in fractured starch granules in barley. Theor Appl Genet. 2018:131(2):353–364. 10.1007/s00122-017-3006-1 29098311

[kiae429-B33] Seung D . Amylose in starch: towards an understanding of biosynthesis, structure and function. New Phytol. 2020:228(5):1490–1504. 10.1111/nph.16858 32767769

[kiae429-B34] Seung D, Boudet J, Monroe JD, Schreier TB, David LC, Abt M, Lu K-J, Zanella M, Zeeman SC. Homologs of PROTEIN TARGETING TO STARCH control starch granule initiation in Arabidopsis leaves. Plant Cell. 2017:29(7):1657–1677. 10.1105/tpc.17.00222 28684429 PMC5559754

[kiae429-B35] Seung D, Schreier TB, Bürgy L, Eicke S, Zeeman SC. Two plastidial coiled-coil proteins are essential for normal starch granule initiation in Arabidopsis. Plant Cell. 2018:30(7):1523–1542. 10.1105/tpc.18.00219 29866647 PMC6096604

[kiae429-B36] Seung D, Smith AM. Starch granule initiation and morphogenesis—progress in Arabidopsis and cereals. J Exp Bot. 2019:70(3):771–784. 10.1093/jxb/ery412 30452691

[kiae429-B37] Smith AM, Zeeman SC. Starch: a flexible, adaptable carbon store coupled to plant growth. Annu Rev Plant Biol. 2020:71(1):217–245. 10.1146/annurev-arplant-050718-100241 32075407

[kiae429-B38] Soh HN, Sissons MJ, Turner MA. Effect of starch granule size distribution and elevated amylose content on durum dough rheology and spaghetti cooking quality. Cereal Chem. 2006:83(5):513–519. 10.1094/CC-83-0513

[kiae429-B39] Tetlow I, Emes M. Starch biosynthesis in the developing endosperms of grasses and cereals. Agronomy. 2017:7(4):81. 10.3390/agronomy7040081

[kiae429-B40] Uauy C, Wulff BBH, Dubcovsky J. Combining traditional mutagenesis with new high-throughput sequencing and genome editing to reveal hidden variation in polyploid wheat. Annu Rev Genet. 2017:51(1):435–454. 10.1146/annurev-genet-120116-024533 28934591

[kiae429-B41] Vandromme C, Spriet C, Dauvillée D, Courseaux A, Putaux J-L, Wychowski A, Krzewinski F, Facon M, D'Hulst C, Wattebled F. PII1: a protein involved in starch initiation that determines granule number and size in Arabidopsis chloroplast. New Phytol. 2019:221(1):356–370. 10.1111/nph.15356 30055112

[kiae429-B42] Walkowiak S, Gao L, Monat C, Haberer G, Kassa MT, Brinton J, Ramirez-Gonzalez RH, Kolodziej MC, Delorean E, Thambugala D, et al Multiple wheat genomes reveal global variation in modern breeding. Nature. 2020:588(7837):277–283. 10.1038/s41586-020-2961-x 33239791 PMC7759465

[kiae429-B43] Warren FJ, Gidley MJ, Flanagan BM. Infrared spectroscopy as a tool to characterise starch ordered structure—a joint FTIR-ATR, NMR, XRD and DSC study. Carbohydr Polym. 2016:139:35–42. 10.1016/j.carbpol.2015.11.066 26794944

[kiae429-B44] Watson-Lazowski A, Raven E, Feike D, Hill L, Elaine Barclay J, Smith AM, Seung D. Loss of PROTEIN TARGETING TO STARCH 2 has variable effects on starch synthesis across organs and species. J Exp Bot. 2022:73(18):6367–6379. 10.1093/jxb/erac268 35716106 PMC9578351

[kiae429-B45] Zhou Y, Zhao X, Li Y, Xu J, Bi A, Kang L, Xu D, Chen H, Wang Y, Wang Y-g, et al Triticum population sequencing provides insights into wheat adaptation. Nat Genet. 2020:52(12):1412–1422. 10.1038/s41588-020-00722-w 33106631

