# Supplementary material for: Wheat MYOSIN-RESEMBLING CHLOROPLAST PROTEIN controls B-type starch granule initiation timing during endosperm development
Source: Plant Physiol. 2024 Aug 19;196(3):1980–96. doi: 10.1093/plphys/kiae429 (PMC11531834; doi:10.1093/plphys/kiae429)
Supplement: kiae429_Supplementary_Data [file kiae429_supplementary_data.zip › Supplementary Figures and Tables v2.pdf]

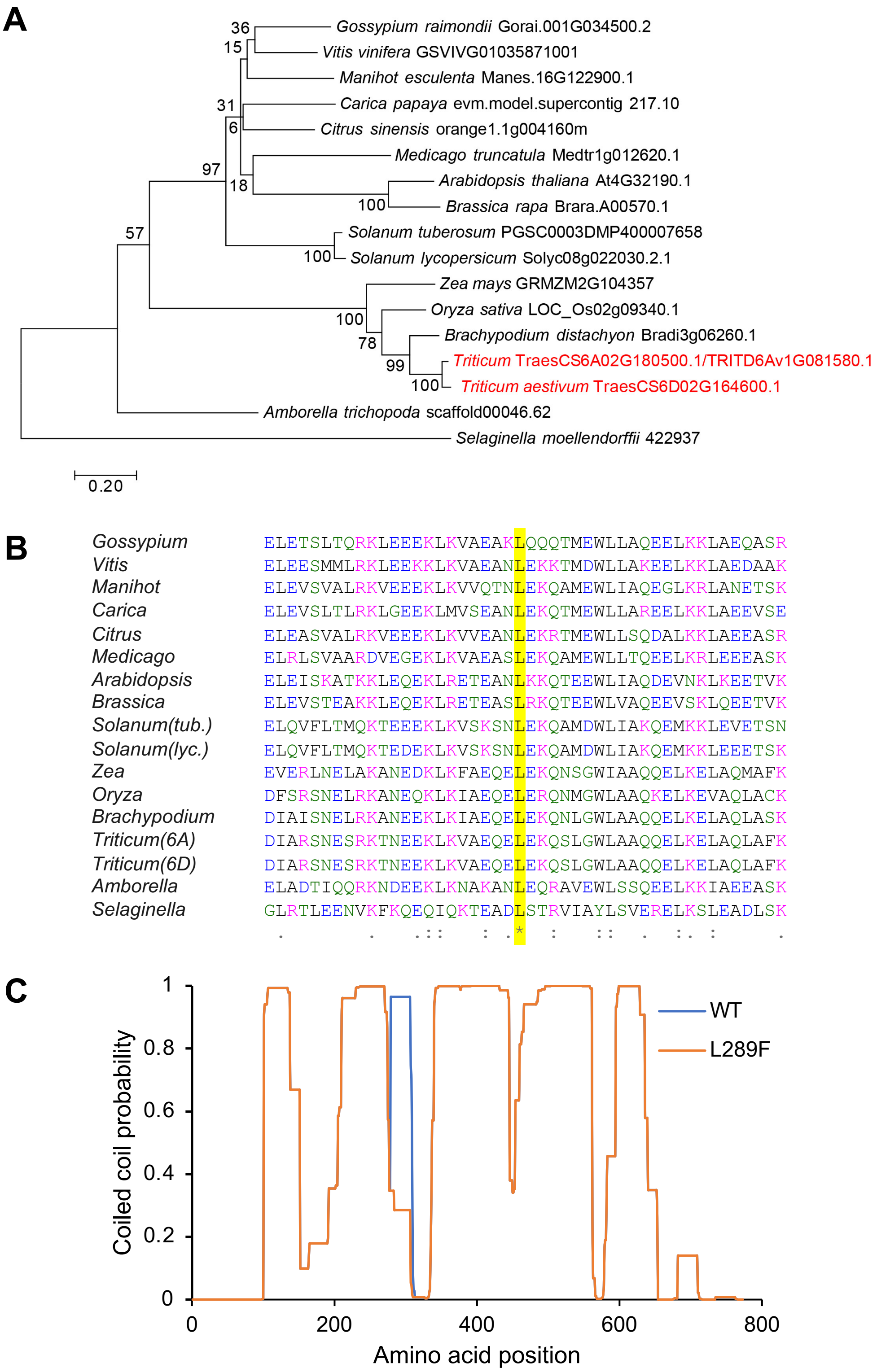


**Supplementary Figure S1. Phylogenetic analysis of wheat MRC sequences. A)** Phylogenetic tree of MRC orthologs. The amino acid sequences of the orthologs identified in Seung *et al.* 2018 were aligned with the wheat sequences. A maximum likelihood tree was assembled with 1000 bootstraps. Bootstrap values are shown next to each node. Branch lengths indicate the number of substitutions per site, as indicated by the scale bar. The wheat sequences are highlighted in red. **B)** Multiple sequence alignment of MRC orthologs generated with Clustal O. The region surrounding Leu289 (highlighted in yellow) is shown. Symbols under the alignment indicate conserved residues with: complete identity (*), highly similar properties (:) or weakly similar properties (.). Colors of residues represent side chain properties: hydrophobic (black), acidic (blue), basic (magenta) and other (green). **C)** Coiled coil prediction of *Ta*MRC-A1 wild type (WT) sequence and the Leu289Phe (L289F) mutant using COILS/PCOILS. The probability was calculated using the 28 amino acid window.

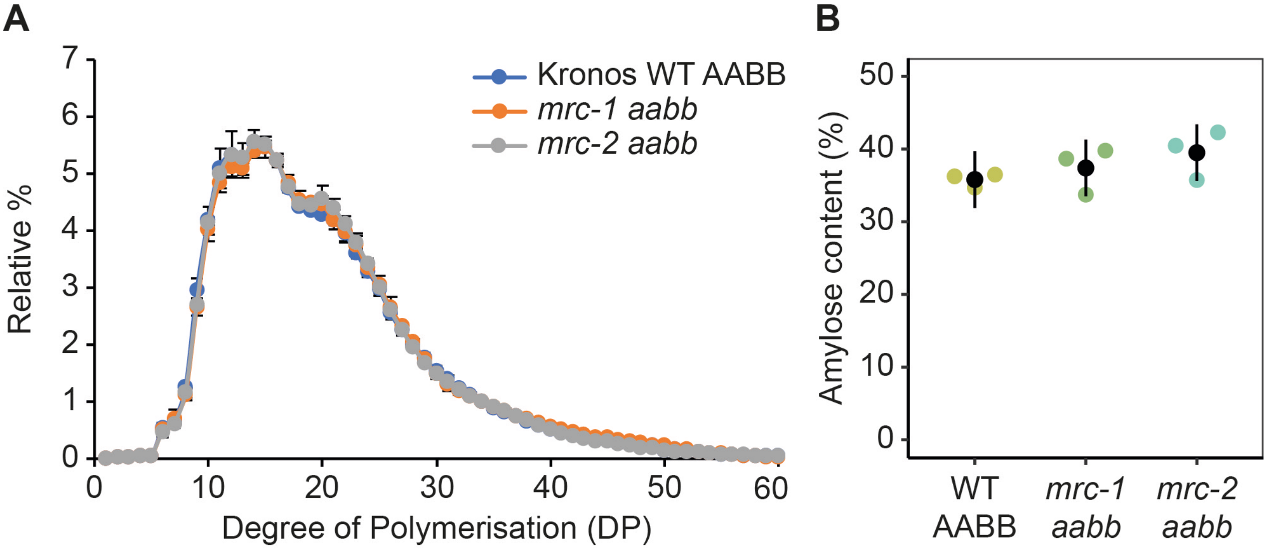


**Supplementary Figure S2. Chain length distribution and amylose content of the *mrc* mutants. A)** Chain length distribution of wild type (WT AABB) *mrc-1 aabb* and *mrc-2 aabb* starch. Purified starch was debranched and analyzed with High Performance Anion Exchange Chromatography with Pulsed Amperometric Detection (HPAEC-PAD). The area of peaks corresponding to chains of each degree of polymerization (DP) was expressed as a percentage of the summed peak area for DP 1–60. Values are the mean ± SEM from three replicate measurements. **B)** Amylose content of WT AA BB, *mrc-1 aabb* and *mrc-2 aabb* starch quantified using iodine colorimetry. Values represent mean ± 95% CI from three replicate measurements.

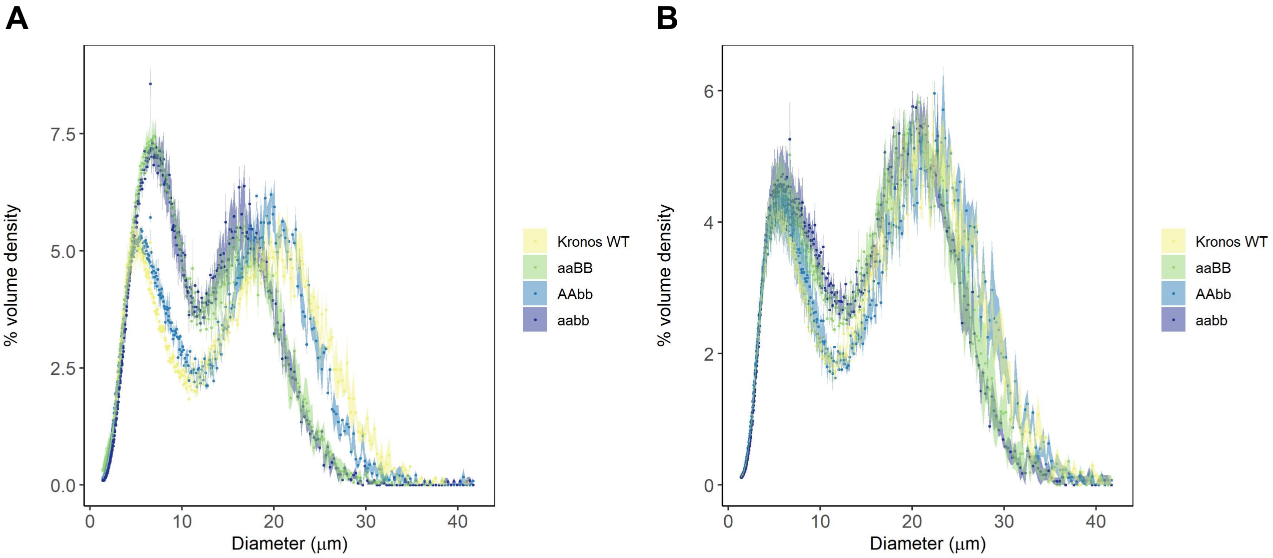


**Supplementary Figure S3. The 6B pseudogene does not contribute to granule size distribution in wheat endosperm starch.** Size distributions were determined by measuring at least 100,000 granules per replicate with a Coulter counter set to total count mode, and are plotted here with evenly binned x-axes. Data points are mean values from 3 individual plants of each genotype (3 grains from each plant), with the standard error of the mean shown as a shaded ribbon. **A)** Starch from the wild type (WT) and genotypes isolated from the *mrc-1* cross: the single A homoeolog mutant (*aa*BB), the single B pseudogene mutant (AA*bb*), and the double mutant (*aabb*). **B)** Same as A), but with genotypes from the *mrc-2* cross.

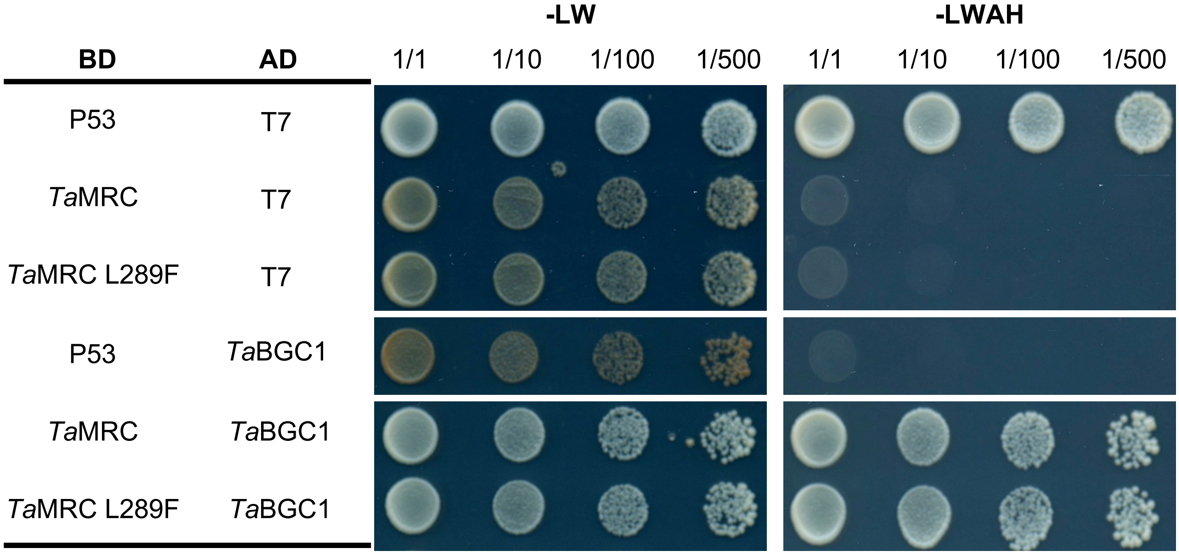


**Supplementary Figure S4. Direct interaction between *Ta*MRC and *Ta*BGC1 in yeast two-hybrid (Y2H).** *Ta*MRC and *Ta*MRC with the L289F mutation with Y2H binding domain both result in yeast growth when paired with *Ta*BGC1 with Y2H activation domain. P53 = murine P53 control, T7 = V40 large T-antigen control. P53-T7 interaction is the positive control, other combinations with P53 and T7 are negative controls. AD = GAL4 activation domain (pGADT7 vector), BD = GAL4 binding domain (pGBKT7 vector). –LW = double dropout medium, without leucine and tryptophan. – LWAH = quadruple dropout medium, without leucine, tryptophan, adenine, histidine. Numbers above images indicate dilution factors: 1/1 = no dilution, 1/10 = 1 in 10 dilution, 1/100 = 1 in 100 dilution, 1/500 = 1 in 500 dilution.

**Supplementary Table S1. Amino acid identity and similarity values between Arabidopsis and wheat MRC.** For each pairwise comparison, identity and similarity values (presented as identity/similarity) were calculated using pairwise BLASTp.

| | ***Ta*MRC-A1** (TraesCS6A02G180500) | ***Tt*MRC-A1** (TRITD6Av1G081580) | ***Ta*MRC-D1** (TraesCS6D02G164600) | ***At*MRC** (At4g32190) |
|---|---|---|---|---|
| ***Ta*MRC-A1** (TraesCS6A02G180500) | | | | |
| ***Tt*MRC-A1** (TRITD6Av1G081580) | 100/100 | | | |
| ***Ta*MRC-D1** (TraesCS6D02G164600) | 95/97 | 95/97 | | |
| ***At*MRC** (At4g32190) | 32/57 | 32/57 | 32/56 | |

**Supplementary Table S2. Reads mapped to genetic signatures of retrotransposon insertion in *MRC-B1.*** *Triticum turgidum ssp. durum* and *Triticum dicoccoides* species (Zhou *et al.* 2020) were aligned to the 'tetraploid' version of the Chinese Spring reference genome, and the number of reads mapped to each of the three genetic signatures in each line is indicated.

| Zhou *et al.* (2020) Line ID | Subspecies | 5' UTR and exon two junction | 5' junction of the retrotransposon insertion | 3' junction of the retrotransposon insertion |
|---|---|---|---|---|
| PI 24493 | Triticum turgidum L. ssp. durum (Desf.) Husn. | 3 | 4 | 2 |
| PI 166327 | Triticum turgidum L. ssp. durum (Desf.) Husn. | 1 | 7 | 8 |
| PI 178143 | Triticum turgidum L. ssp. durum (Desf.) Husn. | 1 | 1 | 7 |
| PI 192051 | Triticum turgidum L. ssp. durum (Desf.) Husn. | 4 | 4 | 2 |
| PI 623461 | Triticum turgidum L. ssp. durum (Desf.) Husn. | 3 | 0 | 6 |
| PI 624129 | Triticum turgidum L. ssp. durum (Desf.) Husn. | 2 | 9 | 8 |
| PI 624388 | Triticum turgidum L. ssp. durum (Desf.) Husn. | 4 | 4 | 4 |
| PI 625273 | Triticum turgidum L. ssp. durum (Desf.) Husn. | 2 | 6 | 7 |
| PI 626483 | Triticum turgidum L. ssp. durum (Desf.) Husn. | 3 | 5 | 0 |
| PI 627514 | Triticum turgidum L. ssp. durum (Desf.) Husn. | 4 | 5 | 6 |
| PI 627942 | Triticum turgidum L. ssp. durum (Desf.) Husn. | 4 | 4 | 5 |
| PI 627996 | Triticum turgidum L. ssp. durum (Desf.) Husn. | 2 | 2 | 3 |
| PI 428016 | Triticum turgidum L. ssp. dicoccoides (Korn. ex Asch. & Graebn.) Thell. | 2 | 4 | 5 |
| PI 466933 | Triticum turgidum L. ssp. dicoccoides (Korn. ex Asch. & Graebn.) Thell. | 4 | 4 | 4 |
| PI 466970 | Triticum turgidum L. ssp. dicoccoides (Korn. ex Asch. & Graebn.) Thell. | 2 | 4 | 0 |
| PI 471062 | Triticum turgidum L. ssp. dicoccoides (Korn. ex Asch. & Graebn.) Thell. | 5 | 3 | 7 |
| PI 487254 | Triticum turgidum L. ssp. dicoccoides (Korn. ex Asch. & Graebn.) Thell. | 3 | 2 | 0 |
| PI 428098 | Triticum turgidum L. ssp. dicoccoides (Korn. ex Asch. & Graebn.) Thell. | 4 | 1 | 1 |
| PI 428138 | Triticum turgidum L. ssp. dicoccoides (Korn. ex Asch. & Graebn.) Thell. | 5 | 8 | 2 |
| TRI 11505 | Triticum turgidum L. ssp. dicoccoides (Korn. ex Asch. & Graebn.) Thell. | 3 | 2 | 4 |
| PI 428041 | Triticum turgidum L. ssp. dicoccoides (Korn. ex Asch. & Graebn.) Thell. | 2 | 2 | 1 |
| PI 428071 | Triticum turgidum L. ssp. dicoccoides (Korn. ex Asch. & Graebn.) Thell. | 3 | 2 | 4 |

**Supplementary Table S3. Pairwise comparisons of wheat growth phenotypes.** Statistical analyses were conducted on the data presented in Figure 3, where the same group of plants were measured for all phenotypes and *n* = 9 – 10 individual plants. For tiller number per plant and grain number per plant, we present ratios of pairwise comparisons between genotypes, based on a Poisson regression model with post-hoc tests performed on the log scale and intervals back-transformed from the log scale. For the post-hoc tests, p-value and 95% confidence intervals were adjusted using Bonferroni method. Statistics were carried out in R, using the glm() function from the 'GLMMadaptive' package. For thousand grain weight and grain size, we present differences of pairwise comparisons between genotypes, based on a linear regression model and one-way ANOVA with Tukey post-hoc test. Statistics were done in R using the lm() function from the 'stats' package. All pairwise comparisons were done using the emmeans() function from the 'emmeans' package. Comparisons where p<0.05 are highlighted in grey. WT = wild type, SE = standard error, 95% CI = 95% confidence interval, df = degrees of freedom.

| **Tiller number per plant** | | | | | | |
|---|---|---|---|---|---|---|
| **contrast** | **ratio** | **SE** | **z.ratio** | **p.value** | **- 95% CI** | **+ 95 CI** |
| WT / *mrc-1* | 0.82 | 0.152 | -1.07 | 1 | 0.48 | 1.41 |
| WT / *mrc-2* | 0.78 | 0.142 | -1.39 | 1 | 0.45 | 1.32 |
| WT / *mrc-3* | 0.91 | 0.173 | -0.48 | 1 | 0.52 | 1.59 |
| WT / *mrc-1* BC2 *AABB* | 0.91 | 0.173 | -0.48 | 1 | 0.52 | 1.59 |
| WT / *mrc-1* BC2 *aabb* | 1.01 | 0.196 | 0.06 | 1 | 0.57 | 1.79 |
| *mrc-1/ mrc-2* | 0.95 | 0.159 | -0.34 | 1 | 0.58 | 1.55 |
| *mrc-1 /mrc-3* | 1.11 | 0.195 | 0.61 | 1 | 0.67 | 1.86 |
| *mrc-1 / mrc-1* BC2 *AABB* | 1.11 | 0.195 | 0.61 | 1 | 0.67 | 1.86 |
| *mrc-1 / mrc-1* BC2 *aabb* | 1.23 | 0.222 | 1.16 | 1 | 0.73 | 2.09 |
| *mrc-2 / mrc-3* | 1.18 | 0.203 | 0.95 | 1 | 0.71 | 1.95 |
| *mrc-2 / mrc-1* BC2 *AABB* | 1.18 | 0.203 | 0.95 | 1 | 0.71 | 1.95 |
| *mrc-2 / mrc-1* BC2 *aabb* | 1.30 | 0.232 | 1.49 | 1 | 0.77 | 2.20 |
| *mrc-3 / mrc-1* BC2 *AABB* | 1 | 0.180 | 1.36E-15 | 1 | 0.59 | 1.69 |
| *mrc-3 / mrc-1* BC2 *aabb* | 1.11 | 0.204 | 0.55 | 1 | 0.64 | 1.90 |
| mrc-1 BC2 *AABB* / mrc-1 BC2 *aabb* | 1.11 | 0.204 | 0.55 | 1 | 0.64 | 1.90 |
| **Grain number per plant** | | | | | | |
| **contrast** | **ratio** | **SE** | **z.ratio** | **p.value** | **- 95% CI** | **+ 95 CI** |
| WT / *mrc-1* | 1.05 | 0.045 | 1.026 | 1 | 0.92 | 1.19 |
| WT / *mrc-2* | 0.91 | 0.038 | -2.335 | 0.293151 | 0.80 | 1.03 |
| WT / *mrc-3* | 1.15 | 0.051 | 3.117 | 0.027373 | 1.01 | 1.31 |
| WT / *mrc-1* BC2 *AABB* | 1.28 | 0.058 | 5.464 | 6.95E-07 | 1.12 | 1.46 |
| WT / *mrc-1* BC2 *aabb* | 1.04 | 0.045 | 1.005 | 1 | 0.92 | 1.18 |
| mrc-1 */mrc-2* | 0.87 | 0.036 | -3.452 | 0.008351 | 0.77 | 0.98 |
| mrc-1 / *mrc-3* | 1.10 | 0.048 | 2.147 | 0.477097 | 0.97 | 1.24 |
| *mrc-1 / mrc-1* BC2 *AABB* | 1.23 | 0.055 | 4.554 | 7.88E-05 | 1.08 | 1.40 |
| *mrc-1 / mrc-1* BC2 *aabb* | 1.00 | 0.042 | -0.021 | 1 | 0.88 | 1.13 |
| *mrc-2 / mrc-3* | 1.26 | 0.053 | 5.583 | 3.55E-07 | 1.12 | 1.43 |
| *mrc-2 / mrc-1* BC2 *AABB* | 1.41 | 0.061 | 7.956 | 2.67E-14 | 1.24 | 1.60 |
| *mrc-2 / mrc-1* BC2 *aabb* | 1.15 | 0.047 | 3.431 | 0.009028 | 1.02 | 1.30 |
| *mrc-3 / mrc-1* BC2 *AABB* | 1.12 | 0.051 | 2.418 | 0.23417 | 0.98 | 1.28 |
| *mrc-3 / mrc-1* BC2 *aabb* | 0.91 | 0.040 | -2.168 | 0.452384 | 0.80 | 1.03 |
| mrc-1 BC2 *AABB* / mrc-1 BC2 *aabb* | 0.81 | 0.036 | -4.575 | 7.13E-05 | 0.71 | 0.93 |

| **Thousand Grain Weight (g)** | | | | | | | |
|---|---|---|---|---|---|---|---|
| **contrast** | **difference** | **SE** | **df** | **t.ratio** | **p.value** | **- 95% CI** | **+ 95 CI** |
| WT - *mrc-1* | 0.597 | 2.768 | 53 | 0.215819 | 0.999932 | -7.588 | 8.782 |
| WT - *mrc-2* | 3.031 | 2.768 | 53 | 1.094812 | 0.881287 | -5.154 | 11.216 |
| WT - *mrc-3* | -5.275 | 2.768 | 53 | -1.90551 | 0.410261 | -13.460 | 2.910 |
| WT - *mrc-1* BC2 *AABB* | -8.684 | 2.768 | 53 | -3.13658 | 0.031545 | -16.869 | -0.499 |
| WT - *mrc-1* BC2 *aabb* | 1.104 | 2.768 | 53 | 0.3986 | 0.998628 | -7.081 | 9.289 |
| *mrc-1 - mrc-2* | 2.433 | 2.695 | 53 | 0.903079 | 0.944029 | -5.533 | 10.400 |
| *mrc-1 - mrc-3* | -5.873 | 2.695 | 53 | -2.17945 | 0.264477 | -13.840 | 2.094 |
| *mrc-1 - mrc-1* BC2 *AABB* | -9.281 | 2.695 | 53 | -3.44427 | 0.013618 | -17.248 | -1.314 |
| *mrc-1 - mrc-1* BC2 *aabb* | 0.506 | 2.695 | 53 | 0.187789 | 0.999966 | -7.461 | 8.473 |
| *mrc-2 - mrc-3* | -8.306 | 2.695 | 53 | -3.08253 | 0.036314 | -16.273 | -0.340 |
| *mrc-2 - mrc-1* BC2 *AABB* | -11.715 | 2.695 | 53 | -4.34735 | 0.000851 | -19.681 | -3.75 |
| *mrc-2 - mrc-1* BC2 *aabb* | -1.927 | 2.695 | 53 | -0.71529 | 0.979257 | -9.894 | 6.039 |
| *mrc-3 - mrc-1* BC2 *AABB* | -3.408 | 2.695 | 53 | -1.26481 | 0.802435 | -11.375 | 4.558 |
| *mrc-3 - mrc-1* BC2 *aabb* | 6.379 | 2.695 | 53 | 2.367244 | 0.186556 | -1.588 | 14.346 |
| *mrc-1* BC2 *AABB - mrc-1* BC2 *aabb* | 9.787 | 2.695 | 53 | 3.632056 | 0.007919 | 1.820 | 17.754 |

| **Grain size (area mm²)** | | | | | | | |
|---|---|---|---|---|---|---|---|
| **contrast** | **difference** | **SE** | **df** | **t.ratio** | **p.value** | **- 95% CI** | **+ 95 CI** |
| WT - *mrc-1* | -0.684 | 0.668 | 53 | -1.02467 | 0.907566 | -2.658 | 1.290 |
| WT - *mrc-2* | 0.279 | 0.668 | 53 | 0.418244 | 0.998272 | -1.695 | 2.253 |
| WT - *mrc-3* | -1.951 | 0.668 | 53 | -2.92194 | 0.054466 | -3.925 | 0.0231 |
| WT - *mrc-1* BC2 *AABB* | -2.077 | 0.668 | 53 | -3.11028 | 0.033791 | -4.051 | -0.103 |
| WT - *mrc-1* BC2 *aabb* | -0.809 | 0.668 | 53 | -1.2119 | 0.82914 | -2.783 | 1.165 |
| *mrc-1 - mrc-2* | 0.964 | 0.650 | 53 | 1.482452 | 0.676692 | -0.958 | 2.885 |
| *mrc-1 - mrc-3* | -1.267 | 0.650 | 53 | -1.94926 | 0.384721 | -3.188 | 0.655 |
| *mrc-1 - mrc-1* BC2 *AABB* | -1.393 | 0.650 | 53 | -2.14276 | 0.281888 | -3.314 | 0.529 |
| *mrc-1 - mrc-1* BC2 *aabb* | -0.125 | 0.650 | 53 | -0.19236 | 0.999961 | -2.046 | 1.797 |
| *mrc-2 - mrc-3* | -2.230 | 0.650 | 53 | -3.43171 | 0.01411 | -4.152 | -0.309 |
| *mrc-2 - mrc-1* BC2 *AABB* | -2.356 | 0.650 | 53 | -3.62521 | 0.00808 | -4.278 | -0.435 |
| *mrc-2 - mrc-1* BC2 *aabb* | -1.089 | 0.650 | 53 | -1.67481 | 0.55407 | -3.010 | 0.833 |
| *mrc-3 - mrc-1* BC2 *AABB* | -0.126 | 0.650 | 53 | -0.1935 | 0.99996 | -2.047 | 1.796 |
| *mrc-3 - mrc-1* BC2 *aabb* | 1.142 | 0.650 | 53 | 1.756897 | 0.501633 | -0.780 | 3.063 |
| *mrc-1* BC2 *AABB - mrc-1* BC2 *aabb* | 1.268 | 0.650 | 53 | 1.950401 | 0.384065 | -0.654 | 3.189 |

**Supplementary Table S4. KASP markers for genotyping the wheat mutants.** All primer sequences are given 5' to 3'. The wild-type allele primers had the VIC/HEX tail (GAAGGTCGGAGTCAACGGATT) on the 5' ends, while the mutant allele primers had the FAM tail (GAAGGTGACCAAGTTCATGCT) on the 5' ends. The nucleotide(s) that discriminate the wild-type from the mutated base (for wild-type and mutant primers) or the homoeologous SNPs (for common primers) are indicated in capital letters.

| Gene | Line and mutation | Wild-type allele | Mutant allele | Common |
|---|---|---|---|---|
| *Tt*MRC-A1 | Kronos3272 (Q258*) | agcaacagttagggagctgC | agcaacagttagggagctgT | cctcgattcatttgatctggcg |
| | Kronos598 (L289F) | ccatcctaaactctgcttctcaaG | ccatcctaaactctgcttctcaaA | gaagcaacagttaggGAGcT |
| | Kronos4681 (Q550*) | catacggctcagatgctcgC | catacggctcagatgctcgT | gcaagatcgccagtgagC |
| | | | | |
| 6B pseudogene | Kronos4305 | ttgagaagcagagtttaggatgG | ttgagaagcagagtttaggatgA | acgtttgaagtcagtgataataccA |
| | Kronos3078 | aggattcagagctttctgatacaC | aggattcagagctttctgatacaT | tgaagctcaGcaatttcactgc |
