# Supplementary material for: Wheat MYOSIN-RESEMBLING CHLOROPLAST PROTEIN controls B-type starch granule initiation timing during endosperm development
Source: Plant Physiol. 2024 Aug 19;196(3):1980–96. doi: 10.1093/plphys/kiae429 (PMC11531834; doi:10.1093/plphys/kiae429)
Supplement: kiae429_Supplementary_Data [file kiae429_supplementary_data.zip › Supplementary File S1.docx]

**Supplementary File S1. *Aegilops speltoides* *MRC* gene model**. The genome of *Aegilops speltoides* isolate TS01 was searched using BLASTn, using the *TaMRC-A1* coding sequence as the query. A match was identified on Chromosome 6 in the region from 180007045 to 180009267, with 97.5% sequence identity. Sequences highlighted in yellow represent the two putative exons of MRC, red text represents the start and stop codons, blue text represents splice junctions.

>CM038146.1:180003045-180012267 Aegilops speltoides isolate TS01 chromosome 6, whole genome shotgun sequence

TAATGGCTCTCTCCACGAATGAAGAGGGGGTGGAGGGGTATAAATAGCCTCCACACAAAATCCAACCGTTACACACATTTGAGCAATCTCGGTGGGACCAAAGTGGACAACACGGTGAGACCGATAGTTCAAATAATGTGAACGTTGGGATTTTTGGTGGGACCGAAAATACAATCTCGGTTAGTCCGATTCGTGCAATGACAAGAGCATGCCAATTTGGTGGGACCGACTTCATCATCTCGGTGACACCGATTTGATGATGTAGGTTCTGAAGCTTGACAACTCATGTTTGGTGAGACCGAGTTCGTAATCGAAAATCCGAGTTTTCTAGGGTTTGGCTTATGGTGAGGTGGTTCATCTCGGTGTGATTGAGTTTGGAATATCGGTGGGACCGAGACTACTTATGAGTGTATATGGCGGTGAAAGTTCTTGATAGTTTAGGAGATGTATCACTAAGCACTTGAGCAACAGATCATCATCATAACCTCATCCCTTTTAATAGTATTGGCTTTTCCTATGGACTCAATGTGATCTTGGATCACTAAACCAAAAATGGAGTCTTGAGCTTTTGCCAATGCTTGCCTTAGCATTTTGGGGGTCCACTTCTTGATCCTTGCCATACCCATATCAATGAACTCTCTGAAATGATTCTCAAGTAACTATTAGTTCAATGATATATATGTTGTTATTAATTACCAAAACCACTGGGATTAGTTGCACTTTCGGTTTGTAAGTTGGTGCGTTCTAAACAAAGCGTCGTGGCGGGATCTACATGTGAAGCGAGTACATAGCTGCTTCGAAGCAGCAAATGAAGGAGTGCGGATGAAGGAGTTCATTTCGATCTAGGTGTCATACCTAGTGCATCGGGACCAATGAAGATCTTCGTGACAATCTTTGGTGCAATTGCCTTGGCAAAAGAATCCAGATTTTACAAGAGGACCAAGCACATCAAGAGACGCTTCAATTCCATTCGGGACCAAGTCCTAGTGGGAGACATAGAGATTTGCAAGATACATACGGATCTGAATGTTGCAGACCCGTTGACTAAGCCTCTCTCACGAGCAAAACATGATTAGCACCAAGACTCCATGGGTGTTAGAATCATTACTATGTAATCTAGATTATTGACTCTAGTGCAAGTGGGAGACTGAAGGAAATATGCCCTAGAGGCAATAATAAAGTTATTATTTATTTCCTCATATCATGATAAATGTTTATTATTCATGCTAGAATTGTATTAACCGAAAACATGATAGATGTGTGAATACGTAGACAAACATATAGTCACTAGTATGCCTCTACTTGACTAGCTCATTAATCAAAGATGGTTATGTTTCCTAACCATAGACATGTGTTGTCATTTGATTAATGGGATCACATCATTATGAGAATGATGTGATTGACATGACCCATTCCGTTAGCCTAGCACTTGATCGTTTAGTATATTGCTATTGCTTTCTTCATGACTTATACAAAGTTCCGCAACTATGAGATTGTGCAACTCCGTTTACGGAAGAACACTTTGTGTGCTACCGGACTCACAACGAATCGGGTGATTATAAAGGTGCTCTCTGGTGTTTAGAAGGTACATGTTGGGTTGGCATAATTCGAGATTAGGTTTTGTCACTCCGATTATCGGAGAGGTATCTCTGGGCCCTCTTGGTAATACTCATCACCTAAGCCTTGCAAGCATTGTAACTAATGAGTTAGTTATAAGATGATGTATTACGGAACGAGTAAAGAGACTTTCCGGGAACGAGATTGAACTAGGTATTGGATACCGACGATCGAATCTCGGGCAAGTAACATACCGATGACAAAGGGAACAACGTATGTTGTTATGCGGTTTGACCGATAAAGATCTTCGTAGAATATGTACGAACCAATATGGGCATCCAGGTCCCGCTATTGGTTATTGACCGAGAATGGTTCTAGGTCATGTCTACATAGTTCTCGAACCCGTAGGGTCCGCACACTTAACGTTACGATGACAGTTTTATTATGAGTTTACAAGTTTTGATGTACCTAAGTTTGTTCGGAGTCCCAGATGTGATCACGGACATGACGAGGAGTCTCGAAATGGTCGAGACATAAAGATTGATATATTGGACGACTATATTCGGACACCTAAGTGTTAGGGTGATTTTGAGAAAATCGGAGTTGGGAGGGTTACCGAACCCCCGGAGAGTATTGGGCCTTATGGGCCTTAGGGAAAGGAGAGAGGGCGGCCAAGATGGGCCGCGCGCCTCCCCTCCGGGTCCGAATTGACTAGAGGGGGTGGCGCTTCCTTTCCTTCCCCCTTCCCTCCTAGTAGGAGTAGGAAAGGAGGAGTCCTACTCCTACTAGGAGGAGGATTCTCCTCCTTGGCACGCCTAAGGGCCGGTGGCCTCCCCTTTGCTCCTTTATATCTCGTGGGCGGGGGGCATAGACACACAAGTTGATCTACGGATCGTTCCTTAGCCGTGTCGGTGCCCCTCCACCATATTCCACCTCGGTCATATTGTCGCGGAGTTTAGGCGAAGCCTGCGTCGGTAGAACATCATCATCGTCACCACGCCGTCGTCTTGTGGAACTCATCTCCGGAGCTTTCTTTGGATCGGAGGCCGAGATCGCCATCGAAATAACGTGTCTTTGAACTCGGAGGCCCGACGTTCGGTGCTTGGATCGGTCGGATCGTGAGGACGACGACTACATCAACCGCGTTGTGTTAACGCTTCGCTTACGGTTCACGAGTGCGTGGACGAACACTCTCCCCTCTCGTTGCTATGCCATCACCATGATCTTGCGTGTGCGTAGGAAATTTTTTGAAATTACTACGTTCCCCAACACCGACATAGAGGAGTCGGCCACCTCCATCCCAAGGATGCAATTTTTTTTTTTGCGGGTGAAGGATGCAGTTCTTCGATAAGAAGTGTTTTCCAATAGCATATGGATGTTGACCTTTTATCCTAGTATTCCATTTCGAGTCAAAATAGTCCCTAACATTATAACAATTTTCATGTGTTACAAACTCCTAACAATTTTCATGTGTTATATAAACTCATTTCTTTTAAGGCAAACAAAGTGTACCTTAAGTTTCATGTGTTATACTCATAAATTTGGAACAGAAAGAAAAAAGGTAGTAAGACGAGTGAACGGGGAAGAAAAGCAGTAGAAGGCAAACGACGCAGCTCTCTCTCACGCTTCTCCCGTGGTCGACGTTGCAGTCCACACGCGGGCACGCGGCTGGGCGCGCCGGTTCCACCACCTCATCTCCCGCACTCCCTCTGCCTCGTATCTCGTCGCCTTCCTCCACACCCCGCAGGAGCATTGCCAGCCGTCCGATCGCGCCCGGGCGGCGGTGGTTCCCTCTCCCCATGTTCCGCGGCC**ATG**CGCCTCTCCATAGGCTCCCCATCCCCGTCGCCGCCGGCGGCGGTGGCCGCCGCTCTCCGCAGCACATCCCCGTCGTGCCGTACCGCCAGTCAT**GT**GAGCGCCCGCTGATCTTTTCTTCCTTTTCTCATATCGCTGTTTCGTGGTACCACGCTGCTCACTGTTACATGGACTGCTCGCGTTCGTGTTTTCCCGATTCCGTGCCCGTCCACACGTGTTTGAAGTAGAAGGATACTAGATTTGGTGTCCTAATTCATGTTCTGCTAGTACTAGTACTTTTTTTAAAAAAACTTTTCTGGAATTGGTTCGATTGTGATAAATTCAGTAAACTGCACCTGGCTGAACAAATCTTGATTGGAGAACGGCCTATGAACTCAAAAAAATTATACTGAACAGATGAAATGTTTATGCAGAGGTATGCTTGAGATCAAATTTCATCGGTTATGATACTTCACCTTATATGACAGTGAATTTCTGAAGTTCAGTGTACTGTCTTTCAGTTCGTCGATTTACAACAATTTTTTACGTGCTTAGTTTGAGGAAAGGATATTCCTCAGATTGCTTCACTAGGTTGTGACCATTTCCTTATCCTAATATCCTACTTATGCATTGTTTCCTGCAACTCTCTC**AG**GTTATGTTCAGGCAGAAGCTGAGTTTTATGGTGGCATTTCAGACTCAGCATCTGAAATATGCTCCTCGCTTGATCAAATCAGCCGTAAAAGGTATTAGATCAAATACAACTGATGGTGATAATGGAACGACTGAGCCAGCTAGAGAGTTGCTGGAGCGGCTATTTGCGAAGACACAAAGTTTAGACACTGGTGCTTCTCATGATAGTGAACTGAGCGTGAGCATTGAGGTCCTGAAGTCTGAATTCGAGGGTGCCTTGTCTATCCTCAGAAACAAAGAGAGGGATCTTCGCAGCGCAGAGAAGAGGGTTTCCGATGATCGGATAAGATTGAGCAAGACGAAGCAGGATCTTGATCAGAGAGAGGAAGCGATCCGCAAAGCTTATGTAAGGCAACAAGGAATAGAGAAAGCACTGAAAAAGGCAAGTAGAGATCTGGCGTTGCGAGTGAAGCAGATCAGTAATCTGAAGCTTTTGGTTGAGGGGCAAGACAGGACTATTGCCAGTTCACAAGCTTTGCTTTCTCAGAAGGTAACTGAAGTGGAAAATCTCAAACGAGATATGTTCAAGAAGAACGAGGAAGCAGACCTGATGCGTTCAGAGATCAGGTCCAAAGAACAGTTGGTTCTTACAGCTAATCAAGCTATTGCGCAGCAAGAAGCAACAGTTAGGGAGCTGCAAAGTGAAATTAAAAGAAAGACGATCGATATCGCCAGATCAAATGAATCGAGGAAAACTAATGAAGAGAAACTGAAAGTTGCTGAACAGGAACTTGAGAAGCAGAGTTTAGGATGGTTAGCAGCACAACAAGAGTTAAAGGAACTTGCACAACTGGCATTCAAAGATACAGATGATATCAATGGTATTATCACTGACTTCAAACGTGTGAGGTCTCTGCTAGATGCTGTACGCTCTGAATTAATCTCTTCAAAAGATGCTTTCGCTTCCTCTCGCAGACAAATAGAAGATCAAGCGGTTCAGTTGCAGGAACAAGTACAGGAACTCGAGGACCAAAGGGTATTACTGATGTCTTACACCCATGATTTGGAGGCTGCTAAACTGGAGATTCAAGGGAAGACACAGGAGCTCAGTTACGCACAGTCTCGTTGTCATGAACTTGAATCACAGTTACTTCAGGAAAGGGAGAAGGTCGAGTCTCTAGAAGCCGAATTAGCCAAAGAAAAACAGAGCTTAGAACATAGAACTGAAGAAGTAGGCTTTCTTCAGAAGGAGCTTGTTCAGAAAGAAAATGAGTGCACCAAATCACAAGAACTTGTTAAAGTAAAAGAGTTTGAGCTGTTAGAAGCCAGACAGGAAGTCCAAGATATGAAGTTAAAGGTAGAGTCTATTCAATTGGCTGTTCAAGAAAAGGATTCAGAGCTTTCTGATACACAGAGCAGACTAACTGAAGTCAGCAGTGAAATTGCTGAGCTTCAGCAGTTGCTAAATAGCAAGAAGGATCAACTGCTTCAGGCTAGAACTGAATTAGATGATAAAGAGCAACATATAGAAACACTGGAGAGTGAGTTGGATAGCATACGGCTCAGATGCTCGCAAGCTGAATCCATGGTTCAAAGGATGGCTGATCTCACTGGCGATCTTGCTAGTTCCGTAAAAGCCGGAGAAATGGACATCTATACATTACTGGATGATGAAATTTCAAGCACTGGTACAGCCCTCGAGTCCAATTTGCATAAGCATAATCAACTGGAGGCTGACATAGAGATGTTAAGAGAATGCTTGCGGCATAAGGACATGGAGTTGAGAGCTGCTCATGAAGCACTTGATGCCAAAGATCACGAGCTGAAGGCAGTACTTAGAAAGTGGGATGTGAAGGAGCGGGAAGTACGTGAGTTAGAAGAGTTACCGGATCCCAGTGCCACAAATGAACTTGCTGGTTTTTCCAGTGAGACAACAGAGGACGGCATTGTAGGAGAGATGGAGCTCCCAGAGCTTCAAATTGAAGCTGTGGAGGTCGAAGCACTTGCTGCTACGACTGCATTGAGGAAGCTAGCGGATATGACTAAGGATTTCTTCAAACACGGCAAAGCTGATTCTGGTATTGACTTGGTTGCATCAGAGAGTCAGAAAATCAGTAAATGTGATCCTAAAATGGAAGTACACAAGAAGACGGATGTGATTCTTGAAGCTGAAAAAGAAATAGTTAGGCTCTTCTCATTGACAAAACAGATTGTCACTGATGACATAATAAACGATATTGAGGAA**TGA**TAGCTTCAAACTGAAGCATGTAGTCTTCCAATTCTATCAAGATAGCTTCAGAGTAGAGATATACCAGATTGATCTTTCGAACATTTATGGACAGTGATGTCGCCCAGAAGGATGAGATCTTCTCTGGTTGATATCACAACTGCCATTTTGAAAAAGGGTAACATGTTGAGCAGAAGCTGGTCATCTGATCCTTTGTTCTCCTTTTGTAATGTACCTCAAACTATTCCTCAGATCTTTGTTCAATGTGTTCCTCCTAAATATACATGGGAAGTTATTGATATGGCAATTGGTGCTGGTTTTCGTCATGCCAAAACTACTACTGAGATTGTGGTGAATGTTTCCATGTGCAGCAAAGTTGGTGCAGCGATGTTGCAGCGTGTTTGTTATTTTACTGTTTCATTGCAACTTCAAATAGTTTCAAATCCCAAAGCTACTAAAGTAGATGGCTATCAAGTACTCTCTCTTTTTTCTTCAAAAAAGGTTAAATACTCTTGTCTTTCCACTTGCTTTCGAAAGCTCCGTAGTTCAAATTTACACAGGATGCTTGAGCAATTTACACAGGATGCTTGAGCATCAAACTGTTAGAAACAATGGGCCTAGTCCATGTACAATTTCTGAAATCTCAAATAGAGACCCATAATTAAAGGGATAATTAGATTTATGCCCCTAGTTGTGTCTCACTCAGCTGTTTTACCCCTAATTTCCAAGAGCCACCGGCTCTCTCCAAGTCACTTCGCTCCTCTTATACTTTTGCCATTTGACCGTTTGATCTTCAGTTTGAAAACCTCATAACTAATTCATACTAAATCAAAAAAATGCAAATAAGATTTCAAAATGTTCGGAAAAACATCACCTATGTGTCAGTGTCATTTGCATTCATGAAAAAAGTGTTGGAAAGTGCACATCTGAGTTTTAGCTCTTATGATACCACCATGAATAGTAAAATGTAGAAAAAAAAAATCAAAAAATTCAAAAACAAATTTGGTGGCAAAGAATGACAAATGTTGTAAGTGCTTGCCAAGTTTTATCAGGGAATGGCATCCGTGGATGTCGTCGCAAAAAAAAATCAGCACTCCAAAATAGATTTTTTTTTGCCACGACTTTCACGAATGTCGTTCCCTCATGAAACTTGGCAAGCACTTAAAACATTTGTCATTCTTTACCACCAATTTTTTTGAATTTTTTTATTTTTTTTTAAATTATACTATTCATGGTGGTATCATAAGAGCTAAAACTCGGATGAGCACTTTCCAACACTTTTTCATGAGTGCAAATGACAATGGCATATAGGTGATGTTTTTCCGAACATTTTGTTATCTTATTTGCATTTTTTTATTTAGTATGAATTAGTTATGAATTTTTCAAACTGATGGTCAAAGGTCATAGGGCAAAAGCATAAGAGGAGCAAAGTGACTTGGATAGAACCGGTGACTTTTGGAAATTATGGGTAAACAACCGAGTGGGTCACAACCAGGGGCATAAATCTAATTATCCCATAATTAAATGGCAAGTGGTGGTGCTAAAGTTTAGAGGAAGTGTTTCACCGCTTTGTATAGTGGGTTCTCTTTTTTTTCGAGAAACTTCCAATCTATTCATTTTCAATCATGGCAGTACAACGAACACCAGAAATAAAAATAATTACATCCAGATTCGTAGACCACCTAATGACTACTACAAGCACTGAAGCGAGCCGAAGGCGCGCCACCATCATCGCCCCTCCCTTGTCGGAGCCGGACACAACTTGTTGTAGTAGACAGTCGGGAAGTCGTCGTGATAAGATCGAAAGGATCAACACACCAGAACAGCAACCGCCGCCGATGAAGACAAACTTAGATCGAAAGGATCCAACCTCCACACTAAGTGATGTGTTGAGAAGAGAAATAGAAGACCACACATGCGCTTGCCTTGCCTCGCCTGGCCGTGCACAACTTCCTTTTGTAGTTTAATTTTTTGATGTCTTGGCAGACAAGTTAATTATTTCTTGTGTGGTAAGTATATGACTTAGAAACCAAGTTGGTTTGAGATCGTGATCACGACATGATACCGCTTCTGGTCCGTTACCAGCCGCGGCAAAAGACACAACGAAAACATCTAGGGTTTTGTCACATCTTGCAACTTACACTGCCACAGTAGTCTATTCCATCCCGAACGCCAACGTGCATCGGCGTGCGCGAGAGGAGGTCTCTGGAAGCGTTCGTCCTTGCGATTTTGCACCGGGAGAGGACGAATTAGGTTTTTGGGAAGTGCTCTGCGTGACTGTCCACGTTCTTCATCACGAGTTGTCTTCTGTCCAAGTCGGGCAGCACTACTCATTGTCATCTACAACAACGTTAGCAAAAGATTGTCGTCAACATCATCATCAACAACATCGCTCCTGCAGTAGCTAACGAACAGTACATCCATTGTAATCTGTTCATGTCTCTATTTGTAGTTATTGTTACATATGTGATGATGCCGTGCATGTTATCTGGTTTGTCTAGTATGCTAGATTATTGCATGCTATTTTTTGTAGTACCATTTATGAATTATTTACTGGAATTAATATTGGATTTGCCTAATATTCCAACACAAACGTGTACAAGCTAAGAAACTTGAGGCTAGAGTTTAACTTTGAATCATCGAACCAACTATTTAAATCCATGGCAGACTAAGGCATAGCCTAGTGGTGGGAAGGGGCTGATGCCTTCCCCCCACCCAGATTCAAGGCATGGTACTTGCAAATTGGGTTTGTTGCACCAATTATACTGTAGGGGGTTCTCTTACAGTCTTTCAGTCAAAAAAAAAATATATTTAAATCCATGCCGAAGAAACTCATGGTCTTGATCATTCTAGCACGCATATAAGTGGTTAACAAAAAAGCACGTCGTCATCTCTTCGGAGACTATTGATATCGATAAATGTGACTCAAAAATGGAAGTACACAAGAACATGGTGATCAGACATTGTAGCCCCGTGTTGTCACCTCGACAAGGCGACAGTGACAACAGGGTTGATCAA
