# Supplementary material for: Wheat MYOSIN-RESEMBLING CHLOROPLAST PROTEIN controls B-type starch granule initiation timing during endosperm development
Source: Plant Physiol. 2024 Aug 19;196(3):1980–96. doi: 10.1093/plphys/kiae429 (PMC11531834; doi:10.1093/plphys/kiae429)
Supplement: kiae429_Supplementary_Data [file pp.00411.2024-s07.pdf › pp.00411.2024-s07.pdf]

Plant Physiology®

# Wheat MRC controls the timing of B-type granule initiation during early endosperm development

Jiawen Chen, Yi Chen, Alexander Watson-Lazowski, Erica Hawkins, Elaine Barclay, Brendan Fahy, Robin Denley Bowers, Kendall Corbin, Frederick Warren, Andreas Blennow, Cristobal Uauy, and David Seung

**Review Timeline:**

| | |
|---|---|
| Submission Date: | 19-Mar-2024 |
| Editorial Decision: | 20-Apr-2024 |
| Revision Received: | 17-Jul-2024 |
| Accepted: | 18-Jul-2024 |

20-Apr-2024

Dr. David Seung
John Innes Centre
Norwich
United Kingdom

RE: Wheat MRC controls the timing of B-type granule initiation during early endosperm development

Dear Dr. Seung:

We have received reviews of your manuscript, which you may view at the end of this letter. On the basis of the advice received and my own evaluation, I believe that, pending revision, this article may ultimately be acceptable for publication in Plant Physiology.

Both reviewers are positive about your manuscript, but raised a number of points that should be carefully addressed during revision. As mentioned by reviewer 1, Y2H alone is not sufficient to conclusively demonstrate a protein-protein interaction and should be backed up with a different method.

When you are ready to submit your revision, click the below link and find the red arrow under your Author Tasks heading for this revision submission.

If you want to visit this version of the manuscript, you may click the link below and then click on the folder that reads "Post Decision Manuscripts".

Link Not Available

When uploading the revised version of this article, please be sure to include as complete an answer as possible to the referees' criticisms during the first round of reviews in the "response to reviewers" section.

Plant Physiology considers a revision for up to 60 days after a manuscript has been provisionally accepted; limited extension of this timeframe is at the discretion of the Monitoring Editor. A reminder notice will be sent to you 2 weeks before the deadline, at which time you may choose to contact the editorial office to request an extension; if so, please provide a justification for your request. After the expiration of this deadline, the article would need to be submitted as a resubmission and would be handled as such.

Thank you very much for giving us an opportunity to review this work. I look forward to receiving the next version.

Sincerely,

Editor, Plant Physiology

----

Want to add this revision deadline to your calendar? Click below!

------------------------------------------------------------------------------ Reviewer comments:
Reviewer #1 (Comments for the Author):

This study generated mutants of a tetraploid wheat that lack function of the protein MRC, and uses those lines to examine the role of MRC in starch biosynthesis in endosperm tissue. MRC was first identified in Arabidopsis by protein-protein association with either PTST2 by the senior author of the current study or with starch synthase 4 by a separate research group. mrc mutants were previously characterized in Arabidopsis leaf, with the finding that they accumulate only one or zero starch granules per plastid instead of the normal number of 5-7, and that single granule is much enlarged. This suggests that MRC is required for starch granule initiation, likely through interaction with ss4 with which it interacts and which causes a similar phenotype in the

mutant condition. This important function, likely to be applicable in determining starch content and quality, justifies continued investigation of MRC specific functions. The study under review here asks if MRC function is the same in wheat endosperm as it is Arabidopsis leaf, using a genetic approach.

The study begins with defining the MRC genes in wheat, providing insight into the evolutionary history of the family. Surveying genome sequences reveals that the ancestor of the wheat B genome contains MRC intact, but all tetraploids and hexaploids examined have a disrupted form of MRC in the B genome. So extant tetraploids are expected to have intact MRC on the A genome but not the B genome, while extant hexaploids will have intact copies on both the A and D genomes but not the B genome. The MRC gene present on the A genome of T. triticum is the subject of the experiments that follow. All this information is pertinent because, conveniently, tetraploid wheat has only one copy of MRC, thus facilitating genetic manipulation. Nonetheless, the study has introduced a missense mutation into the remnant of the presumed mrc pseudogene on the B genome, generating near congenic lines in which there is definitively only one functional copy of MRC in the non-mutant standard and no functional MRC gene in the experimental mutant line. Overall, the genomics and reverse genetics aspects of the study are elegant, clearly explained, and fully appropriate to create comparable lines that vary only by presence or absence of a functional MRC gene.

The mutant and non-mutant lines do not exhibit substantial variation in plant growth, or grain weight or starch content, or starch structure in terms of linear chain length distribution. The number of starch granules per grain, or per amyloplast, does not vary. This is substantially different than the effects of the mrc mutation in Arabidopsis leaf, and leads to the key advance of the study that MRC functions differently with regard to granule initiation in different tissues and/or species. I support this key finding as a noteworthy advance that justifies publication. From this general finding the study proceeds to show that the key difference between mrc mutants and wild type is the timing of initiation of B type starch granules, the small ones. Normally these appear in wild type at about 20 dpa, whereas in the mrc mutant they show up at about 14 dpa (as revealed by the data in Fig. 8). This observation is amply demonstrated by SEM, TEM, and particle size analysis. Also, various mutant lines are examined, i.e., different alleles or backcross derivatives, with repeatable findings. So the overall conclusion that the B granules show up earlier if MRC is not working is well supported.

In comments that follow I detail some confusion of this reviewer in interpreting how the data are explained, in particular the distinctions between the terms "size" referring I think to diameter, and "volume distribution", I think relating to granule shape. This part was hard to interpret for me. and I try to explain in the comments which sections led to my confusion and could perhaps be improved by rewriting targeted to a less immediately informed audience. This does not detract from the central conclusions, but makes the paper somewhat of a chore to follow in some of its sections.

Major comments:

I am thoroughly confused as I try to interpret Fig. 4 relative to Fig. 5. Fig. 4C shows that approximately 95% of the granules are less than 10 μm in diameter, with negligible variation between genotypes. Then Fig. 5A shows a volume distribution that indicates on the x-axis granule diameter and it shows that granules less than 10 μm in diameter make up in the non-mutant line roughly 30% or so of the total and there is a great deal of variation between the genotypes. At first read this suggests a major discrepancy. I think it relates to the fact that the volume of granules less than 10 μm in diameter is much different in the mutant and non-mutant lines, maybe because they are flat in one instance (non-mutant) but spherical in another instance (mrc mutants). Or maybe not. Maybe audiences who are familiar with these plots will know what this all means, but clearly that does not include this reviewer. I think it falls to the authors to better explain what is being measured in Fig. 5. and how Fig. 5 and Fig. 4 are related. As I read this, I see that MRC does not affect the proportion of small granules (lines 252-254) and then that the distribution profiles are very different in wild type and mrc mutants (lines 261-262). Lines 272-275 gets into this, stating that difference are due to granule size because there is no difference in the proportion of small granules, to which I can only say, uh what? Again, "small" refers to granule diameter, and "size" to volume, I think. Again, this has to be explained much more clearly. Overall, the entire section starting on line 256 is very difficult to interpret for this reviewer.

Fig. 11: More confusion here about granule size. The legend suggest a decrease in A type granule size. Are they shrinking? I don't think so. I think this means the relative volume of A type granules decreases as the relative volume of B type granules increases. Again, a better explanation of what is meant by the volume distribution is necessary for this reviewer, who may or may not be indicative of others trying to interpret these data.

Minor comments:

Lines 123-128: It would be useful to state the sequence identity between the query sequence from Arabidopsis and the wheat homologs. This could be an important point, because the main finding is that MRC works differently in Arabidopsis leaf and wheat endosperm.

Lines 777-778: The citation for this paper in the Results indicates 2023, but the listing in the References Cited indicates 2022. The reference should be updated from the preprint to the published version in BMC Plant Biology.

Figure 2: Perhaps add ss4 expression to the plot?

Figure 1B and associated section of Results: It would be useful to explicitly state which alleles are present in the lines analyzed later in the study. I presume that mrc-1 and mrc-2 are homozygous for the nonsense mutation in the otherwise functional mrcA gene and also for the stop codon in the presumed mrcB pseudogene, whereas mrc-2 is homozygous for the missense mutation but contains the non-mutant form of the mrcB pseudogene. This can all be worked out from reading the Results, but I suggest some explicit statements of the genotypes of the plants to be analyzed just to make the genetics easily accessible to the readers.

Yeast two-hybrid: Is it necessary to back this up with co-IP or another type of pull down experiment? Normally Y2H alone is not sufficient to conclusively demonstrate a protein-protein interaction. I realize MRC and BGC1 homologs associate in pull down experiments in Arabidopsis, but should the homologs be tested also in wheat? I leave that to the editors to consider.

Reviewer #2 (Comments for the Author):

Review of manuscript by Chen et al. (Wheat MRC controls the timing of B-type granule initiation during early endosperm development) [MSID: PP2024-RA-00411]

The manuscript by Chen et al. describes work in tetraploid durum wheat (Triticum turgidum) investigating the cellular mechanisms underlying starch granule initiation. Our emerging understanding of the complex pathway of starch granule initiation has been largely defined through studies in the model plant Arabidopsis thaliana L. (Arabidopsis), and the current authors have made important contributions to this body of knowledge in the recent past. The current study in durum wheat aims to elucidate the mechanisms responsible for the bimodal size distribution of starch granules observed in the endosperms of many temperate grasses, in particular those of the Triticaceae. In wheat large A-type granules form at the start of grain filling, from approximately 4 days post anthesis (DPA), followed by formation of small B-type granules around 15-20DPA. B-type granules have been shown to be associated with outgrowths of the plastid envelope membrane termed stromules, although whether B-granule formation requires stromules is not clear. The authors have made the interesting discovery that temporal expression of the gene encoding MYOSIN-RESEMBLING CHLOROPLAST PROTEIN (MRC), known to be involved in starch granule initiation in Arabidopsis, is responsible for the formation of B-type granules later in endosperm development. MRC was shown to be expressed at high levels at early stages of endosperm development, before B-granules form. Specifically, the authors show, using mrc mutants of wheat, that MRC appears to suppress formation of smaller B-type granules early in development, and at later stages of endosperm development when MRC expression is low, B-granules then form. This interesting and novel observation adds important knowledge to our understanding of the many proteins involved in the starch granule initiation pathway, and underlines the importance of studying different plant systems, as the precise role of MRC in Arabidopsis leaves and wheat endosperm is clearly different. The paper is very well written and the work, particularly the gene analysis and genetics, extremely thorough and I look forward to its publication. I have a few comments and minor criticisms that the authors may wish to address, which I hope will improve this very nice piece of work.

1. Figure 5A appears to be duplicated. I'm not sure why the same data for WT and mrc-1 are given twice. I may be missing something, but it is not clear.

2. What about the gene expression of MRC in the wheat leaves? Do the authors propose a similar role for MRC in wheat leaf starch granule initiation as found in Arabidopsis? If the authors have the data, showing MRC expression in leaves would perhaps be an interesting comparison with that of endosperm tissue as shown in Figure 2.

3. The arguments and data in this paper are clear, and very compelling. My only comments on the data regard Figure 9. Please explain the rationale for including the SEM images in Fig.9A, as I'm not sure that they add anything more than has been presented in the previous figures. Likewise, I'm not clear on why the light micrograph sections are presented in Fig. 9B and the TEM sections in Fig 9C. The results section states that the elongated morphology of the amyloplasts in Fig. 9C "...strongly suggests that they are stromules". Based on the images in Fig. 9C I find this interpretation and statement unconvincing. If the authors intend to keep this figure, then I suggest being more cautious in interpreting the images.

4. The model (figure 10) is interesting and helpful, and I agree with the authors that future protein-protein interaction experiments at different stages of endosperm development will prove valuable in determining precise developmental-specific interactions in the granule initiation machinery. In the model, why are stromules only shown at the later stages of amyloplast development when the B-granules are being formed? The prevailing idea is that B-granules form in stromules, however, to my knowledge stromules can form in plastids at various developmental stages and in response to sugars. The authors may wish to keep the message simple in the diagram, but some mention of stromules at earlier stages of development may be worthwhile (unless they know of evidence that they only form when B-granules form in cereal amyloplasts).

5. The discussion describes the biotechnological potential of manipulating the genes involved in granule initiation and morphology determination and is very interesting. However, some discussion of the biological role of MRC I think is warranted. What biological (fitness) advantage does the gene have? Is the expression of MRC and timing of B-granule formation peculiar to

all Triticaceae, or only domesticated grasses? Any insights the authors may have would be a valuable addition to the discussion, I think.

Minor
Reference "Chen et al. 2023" in text (Introduction) is 2022 in references

**Response to Reviewers – Chen et al.**

We thank the editor and both reviewers for their detailed and thoughtful comments, which have greatly improved our manuscript. We have provided responses to all their points below.

**Reviewer 1**

*In comments that follow I detail some confusion of this reviewer in interpreting how the data are explained, in particular the distinctions between the terms "size" referring I think to diameter, and "volume distribution", I think relating to granule shape. This part was hard to interpret for me. and I try to explain in the comments which sections led to my confusion and could perhaps be improved by rewriting targeted to a less immediately informed audience. This does not detract from the central conclusions, but makes the paper somewhat of a chore to follow in some of its sections.*

We thank the reviewer for pointing out the confusion between these terms. We changed the text to better distinguish between granule size, number, and volume. In our study, granule size and number were quantified using a Coulter counter, which measures particle size by quantifying the volume of each particle and is insensitive to the shape of the particle (e.g., spherical, lenticular, etc). Thus, the volumetric size distributions presented in Fig 5A show the percentage (relative to the total volume of measured starch granules) that fall within each size bin. Each size bin is defined by a diameter, which is calculated by the Coulter counter assuming all particles are spherical in shape. Thus, our Coulter counter data encompasses both the size and number of granules, but cannot include any information about starch granule shape. The shape we have only assessed through microscopy. We now clarify these technical details in Lines 259-263.

*I am thoroughly confused as I try to interpret Fig. 4 relative to Fig. 5. Fig. 4C shows that approximately 95% of the granules are less than 10 µm in diameter, with negligible variation between genotypes. Then Fig. 5A shows a volume distribution that indicates on the x-axis granule diameter and it shows that granules less than 10 µm in diameter make up in the non-mutant line roughly 30% or so of the total and there is a great deal of variation between the genotypes. At first read this suggests a major discrepancy. I think it relates to the fact that the volume of granules less than 10 µm in diameter is much different in the mutant and non-mutant lines, maybe because they are flat in one instance (non-mutant) but spherical in another instance (mrc mutants). Or maybe not. Maybe audiences who are familiar with these plots will know what this all means, but clearly that does not include this reviewer. I think it falls to the authors to better explain what is being measured in Fig. 5. and how Fig. 5 and Fig. 4 are related. As I read this, I see that MRC does not affect the proportion of small granules (lines 252-254) and then that the distribution profiles are very different in wild type and mrc mutants (lines 261-262). Lines 272-275 gets into this, stating that difference are due to granule size because there is no difference in the proportion of small granules, to which I can only say, uh what? Again, "small" refers to granule diameter, and "size" to volume, I think. Again, this has to be explained much more clearly. Overall, the entire section starting on line 256 is very difficult to interpret for this reviewer.*

We apologise for this confusion. In typical wheat grains, the small B-type granules make up more than 90% of granules by number, but less than 30% by volume (Kamble et al. 2023; Lindeboom et al. 2004). So even though B-type granules far outnumber A-type granules, they only make up the minority of the total volume of starch since the volume of each individual granule is so small. We

have added this important fact to the introduction to better distinguish between size (volume) and number (Lines 68-70). A change in the relative volume of A- and B-type granules can therefore result from changes in the number of granules, in the size of granules, or in both number and size.

Figures 4C and 5A show different parameters measured from the same set of samples on the Coulter counter. They are distinct in that Figure 4C shows the number of small granules (defined as granules less than 10 µm, so mainly B-type granules) in the different mutant lines, while Figure 5 shows the volumetric size distributions, and values derived from such distributions. The reviewer is correct that mutations in MRC do not affect the proportion of small granules, but this is only when expressed by number (Fig. 4C). When expressed by volume, *mrc* mutations have dramatic alterations in the relative proportions of A- and B-type granules (Fig. 5C). This can be explained by the strong reduction in A-type granule size, and in some lines, the increase in B-type granule size. These differences in size can explain the difference in the volumetric proportions of A- vs. B-type granules, even in the absence of clear changes in the relative number of B-type granules.

We realise that these distinctions were not clearly made in our previous text. We have therefore edited and added text to explain the differences between Figure 4C and 5A, explicitly comparing what is presented in those figures (Lines 265-272). We have also edited the x-axis labels of Figure 4C from “small granule %” to “small granule number %” to highlight that this figure only shows information regarding the relative number of granules within a specific size range, and not the overall distribution of sizes by volume.

*Fig. 11: More confusion here about granule size. The legend suggest a decrease in A type granule size. Are they shrinking? I don't think so. I think this means the relative volume of A type granules decreases as the relative volume of B type granules increases. Again, a better explanation of what is meant by the volume distribution is necessary for this reviewer, who may or may not be indicative of others trying to interpret these data.*

We thank the reviewer for pointing this out. Indeed, the way we worded this in the original manuscript was incorrect. We meant that there was a change in the relative proportion of A-type granules compared to B-type granules in mature grains, as the premature initiation of B-type granules retards A-type granule growth due to the competition for substrates. The A-type granules do not grow and shrink. We have therefore changed the phrase “decrease in A-type granule size” to “reduced growth in A-type granule size” in the figure legend (Line 889-890).

*Lines 123-128: It would be useful to state the sequence identity between the query sequence from Arabidopsis and the wheat homologs. This could be an important point, because the main finding is that MRC works differently in Arabidopsis leaf and wheat endosperm.*

We have added a new Supplemental Table 1 which presents identity and similarity values between the Arabidopsis sequence and the different wheat sequences, and also added in a sentence about this in the main text (Lines 128-130).

*Lines 777-778: The citation for this paper in the Results indicates 2023, but the listing in the References Cited indicates 2022. The reference should be updated from the preprint to the published version in BMC Plant Biology.*

We thank the reviewer for catching this, and we have corrected this citation (Lines 938-940).

*Figure 2: Perhaps add ss4 expression to the plot?*

This is an excellent suggestion. We have now added *Tt*SS4 expression data into the plot.

*Figure 1B and associated section of Results: It would be useful to explicitly state which alleles are present in the lines analyzed later in the study. I presume that mrc-1 and mrc-2 are homozygous for the nonsense mutation in the otherwise functional mrcA gene and also for the stop codon in the presumed mrcB pseudogene, whereas mrc-2 is homozygous for the missense mutation but contains the non-mutant form of the mrcB pseudogene. This can all be worked out from reading the Results, but I suggest some explicit statements of the genotypes of the plants to be analyzed just to make the genetics easily accessible to the readers.*

We thank the reviewer for raising this important point. We have now clarified the genotypes by indicating the alleles present (notated as AABB, aaBB, AAbb, aabb) for all lines in all figures. Previously we only had this allele information for the *mrc-1* backcrossed lines. Full descriptions on what these notations mean are described in Lines 215–221.

*Yeast two-hybrid: Is it necessary to back this up with co-IP or another type of pull down experiment? Normally Y2H alone is not sufficient to conclusively demonstrate a protein-protein interaction. I realize MRC and BGC1 homologs associate in pull down experiments in Arabidopsis, but should the homologs be tested also in wheat? I leave that to the editors to consider.*

We thank the reviewer for raising this. We have dedicated time and resources to provide additional evidence for this interaction, but unfortunately, we have failed due to the recalcitrance of the wheat MRC protein. Firstly, we tried a pairwise co-IP between BGC1 and MRC proteins expressed in *Nicotiana benthamiana* leaves. However, we failed to get robust expression of MRC in this system, despite trying many different tags (YFP, RFP and HA). We also tried to produce an MRC recombinant protein expressed and purified from *E. coli*, but again we tried three different constructs and failed to obtain good protein expression. This has also precluded us from producing a reliable MRC antibody for doing co-IP experiments from wheat extracts. In general, the recalcitrance of wheat MRC to heterologous expression has greatly inhibited further biochemical work on the protein.

However, we also agree with the reviewer that the MRC-BGC1 interaction has been shown for the Arabidopsis orthologs, as well as for the potato orthologs in a study from our lab that is now in revision at another journal. We are therefore not demonstrating a new interaction, but rather showing that the wheat orthologs can also interact. While the interaction was never a novel finding in our study, we still believe that our standalone Y2H experiment with wheat proteins is valuable to readers in the field, as it is an additional independent confirmation of this previously observed interaction. However, we also agree that standards need to be upheld, and it is convention that at least two different methods should be used to conclude a protein-protein interaction.

On balance, we have decided to remove the Y2H results as a main finding of this paper. It has been removed from the main figures and abstract, and moved to a new Supplemental Figure 4. We now cite previous literature to say that the BGC1-MRC interaction has been established in Arabidopsis, and use this as our primary reason for hypothesising that MRC may control B-type granule initiation

through BGC1. We then cite our Y2H results as providing a clue that the wheat proteins may also interact (Lines 374-390; 465-472). This is followed by explicit caveats that more experimental work is required to conclude that the MRC-BGC1 interaction occurs in the endosperm and is the mechanism by which MRC produces the observed phenotypes. We hope for the reviewer's understanding on this matter, and would be open to other suggestions to handle the issue.

**Reviewer 2**

*1. Figure 5A appears to be duplicated. I'm not sure why the same data for WT and mrc-1 are given twice. I may be missing something, but it is not clear.*

We thank the reviewer for pointing out the lack of clarity. The data for the WT and *mrc-1* lines are indeed shown in both graphs in Figure 5A. The data in both panels were obtained at the same time within the same experiment. However, we found it impossible to show all genotypes onto the same plot. Due to the overlap between the traces, the different genotypes could not be distinguished. Therefore, we decided to split the data, and present WT and *mrc-1* on both plots to allow easy comparison. We have added an extra explanation to the legend to clarify this (Lines 818-821).

*2. What about the gene expression of MRC in the wheat leaves? Do the authors propose a similar role for MRC in wheat leaf starch granule initiation as found in Arabidopsis? If the authors have the data, showing MRC expression in leaves would perhaps be an interesting comparison with that of endosperm tissue as shown in Figure 2.*

These are excellent questions. We did not look at gene expression specifically in the wheat leaves, but we agree this is a great idea. To do this in sufficient detail would be a significant undertaking: it is likely that expression patterns follow diel fluctuations like most other starch-related genes, and there is likely variation across different wheat leaves, as well as along individual leaf blades that represent a developmental gradient from younger to older tissue. We therefore felt this experiment was beyond the scope of our current study, and we wish to focus this study on endosperm starch synthesis. However, we already looked at starch phenotypes in the leaves and found that there was a slight reduction in the number of granules per chloroplast in the *mrc* mutants compared to WT (Figure R1, below). This is again distinct from the Arabidopsis mutant, where one large granule accumulates in most chloroplasts (Seung et al., 2018). We therefore feel a follow-up manuscript on the role of MRC in wheat leaves is warranted.

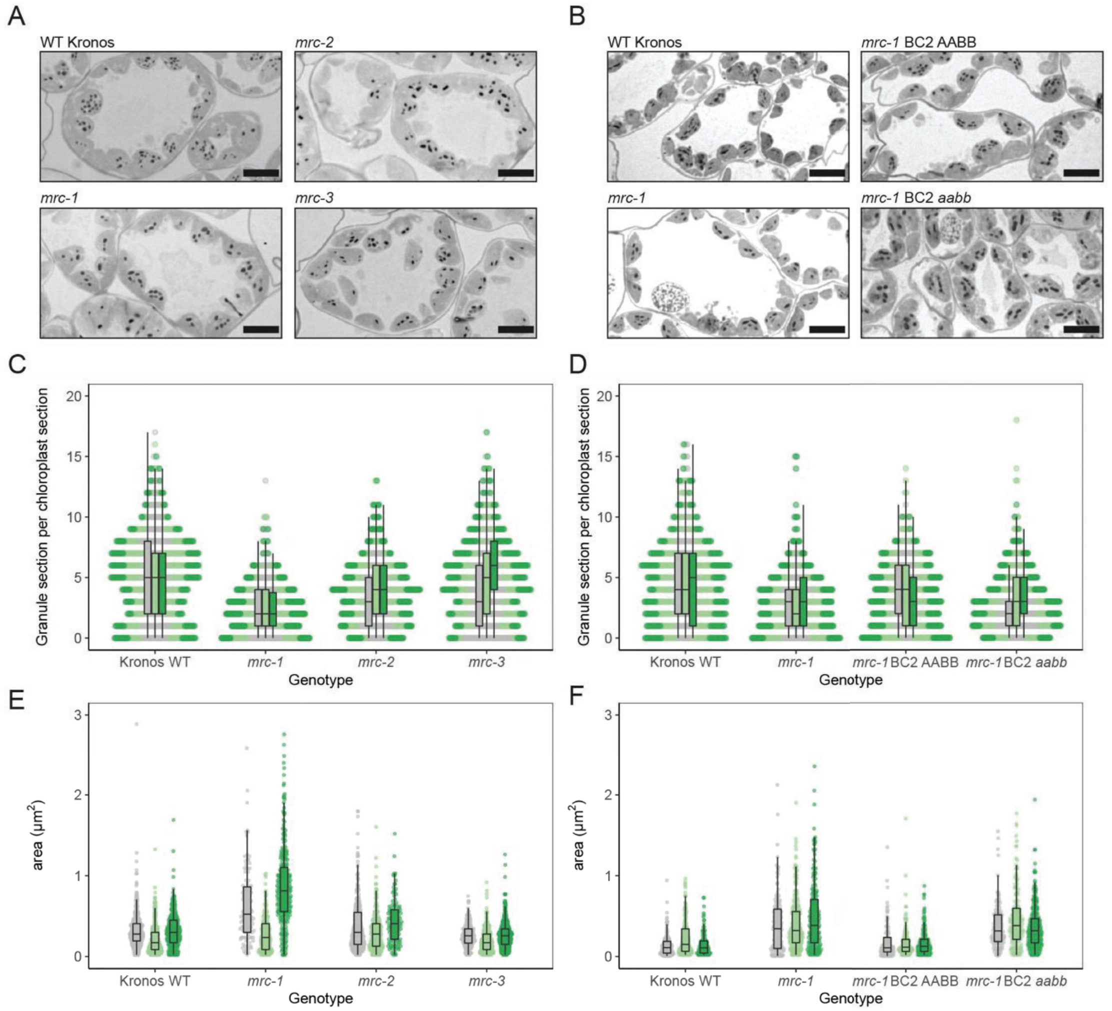


**Figure R1. Chloroplasts of *mrc-1* mutants have fewer and larger granules than the wild type. A, B)** Representative light microscopy images of thin sections (500 nm) from the middle of the older of two leaves in 10-day old wheat seedlings, collected at the end of day. Sections were stained for starch using periodic acid and Schiff staining. Scale bar = 10 µm. **C, D)** Distributions of counts of granule sections per chloroplast section, counted from light microscopy images as in A and B. The three different colours correspond to three individual plants of each genotype, per experiment. For each sample, 200 – 250 chloroplasts were counted, with absolute values varying between samples. Dots represent individual chloroplasts. **E, F)** Distributions of starch granule sizes measured as the area in the light microscopy images as in A and B. The three different colours correspond to three individual plants of each genotype, per experiment. 100 – 400 granules were measured in each sample. Dots represent individual granules. For all boxplots, each box encloses the middle 50% of the distribution, the middle line is the median and the whiskers are the minimum and maximum values within 1.5 of the interquartile range.

*3. The arguments and data in this paper are clear, and very compelling. My only comments on the data regard Figure 9. Please explain the rationale for including the SEM images in Fig.9A, as I'm not sure that they add anything more than has been presented in the previous figures. Likewise, I'm not clear on why the light micrograph sections are presented in Fig. 9B and the TEM sections in Fig 9C. The results section states that the elongated morphology of the amyloplasts in Fig. 9C "...strongly suggests that they are stromules". Based on the images in Fig. 9C I find this interpretation and statement unconvincing. If the authors intend to keep this figure, then I suggest being more cautious in interpreting the images.*

We have carefully considered this point. B-type granule initiation occurs via a distinct spatiotemporal pattern during grain development. Our intention with Figure 9 was to explore the spatial aspect, since the rest of the manuscript focuses on the temporal aspect. The difference between the EM images in Figure 9 and the microscopy in Figure 6 is that it features developing grains, rather than mature grains. In mature grains, it is difficult to examine the locations of B-type granule initiation because the grain is packed full of starch and structural features such as cell walls and amyloplasts are difficult to visualise. Figure 9 shows no difference in the spatial arrangement of A- and B-type granules in the mutants. Figure 9C also highlights how the B-granules are not arranged differently within amyloplast membranes in WT compared to *mrc-1*, suggesting MRC does not influence the spatial organisation of B-type granules within the amyloplast. We have clarified the rationale behind this figure in the text (Lines 352-359). We have also taken aboard the comment about the stromules being unconvincing. The protrusions observed strongly resemble stromules, but the reviewer is correct that without 3D information, this is difficult to conclude. Our main point of Figure 9C is that between WT and *mrc-1*, there were no striking differences in the location of B-type granules (for example, amyloplasts only containing B-type granules, etc). We have edited the text to be more cautious. Now we say that we did not observe anything unusual about the location of B-type granules in the *mrc-1* mutant, rather than making explicit conclusions about stromules (Lines 364-367).

*4. The model (figure 10) is interesting and helpful, and I agree with the authors that future protein-protein interaction experiments at different stages of endosperm development will prove valuable in determining precise developmental-specific interactions in the granule initiation machinery. In the model, why are stromules only shown at the later stages of amyloplast development when the B-granules are being formed? The prevailing idea is that B-granules form in stromules, however, to my knowledge stromules can form in plastids at various developmental stages and in response to sugars. The authors may wish to keep the message simple in the diagram, but some mention of stromules at earlier stages of development may be worthwhile (unless they know of evidence that they only form when B-granules form in cereal amyloplasts).*

We have been studying stromule formation in wheat endosperm using an amyloplast reporter line that expresses mCherry in the plastid stroma (Esch et al., 2023). Currently, we have no evidence of stromules forming in wheat amyloplasts in the absence of B-type granules. We agree that in other plastid types such as chloroplast, stromules can form in response to many triggers. However, we do not know whether the stromules that form in chloroplasts are of the same mechanistic and evolutionary origin as the ones that facilitate B-type granule initiation. We have therefore decided that drawing the model without stromules when the B-type granules have not formed yet is the most representative of what we know about this topic.

*5. The discussion describes the biotechnological potential of manipulating the genes involved in granule initiation and morphology determination and is very interesting. However, some discussion of the biological role of MRC I think is warranted. What biological (fitness) advantage does the gene have? Is the expression of MRC and timing of B-granule formation peculiar to all Triticaceae, or only domesticated grasses? Any insights the authors may have would be a valuable addition to the discussion, I think.*

We thank the reviewer for this suggestion and agree that this is a very interesting point. Understanding the adaptive advantage of different granule types, and thus the mechanisms controlling them, is a major goal of our research. At present we do not know what fitness advantage MRC provides, especially since we also do not know the biological significance of having A- and B-type granules in the first place. We hope to find out more in the future about the adaptive significance of A- and B-type granules and the genes involved in regulating their formation, such as MRC, using the mutants we have generated as a resource. Having a look at MRC expression patterns in wild Triticeae is also a great idea, and we are currently developing the resources to do this. Different species also have different dynamics of grain filling, so it will be interesting to see how the temporal regulation of MRC varies among species that vary in grain filling time. We have added in a paragraph in the discussion to highlight the point about biological significance (Lines 503-514).

*Reference "Chen et al. 2023" in text (Introduction) is 2022 in references*

We thank the reviewer for pointing this out. We have corrected this error.

18-Jul-2024
Dr. David Seung
John Innes Centre
Norwich
United Kingdom

MSID: PP2024-RA-00411R1
MS TITLE: Wheat MRC controls the timing of B-type granule initiation during early endosperm development

Dear Dr. David Seung:

I am pleased to inform you that your revised manuscript "Wheat MRC controls the timing of B-type granule initiation during early endosperm development" has been accepted for publication in Plant Physiology. Your manuscript will be checked for consistency with journal formatting and image standards by one of our Science Editors, who will contact you shortly. They may have a number of queries and suggestions for you. Please keep in mind that they work to increase the online visibility of your article and its attraction for readers from the widest of backgrounds. You will have the opportunity to review any revisions, and I hope you will work constructively with them to get the most out of your research. You will also have the opportunity to review all changes when you receive the author proofs for your article.

Once validated for publication, your article will be sent to a Science Editor for a final review of your manuscript and source files. After your final files are uploaded, your article will appear in the next available issue and the online Preview version of your article will be posted after your signed Copyright and Checklist forms and final source files are received.

Please be sure to include the following in your manuscript file:
Harvard style citations
List of all author contributions and funding information at the beginning of manuscript after the titles and author list.
List all supplemental materials associated with the article, with titles, at the end of the ms, before the references and acknowledgments section.
Complete Figure Legends for all figures appearing in the article listed before the Literature Cited.

Thank you and congratulations on your Plant Physiology paper!

Sincerely,
Editor, Plant Physiology

=====
FIRST AUTHOR PROFILES
=====

We recognize the hard work that goes into being First Author of a Plant Physiology paper by publishing a "First Author Feature" for each First Author alongside your paper.

If the first author(s) would like to submit information for a feature on Facebook and Twitter, fill out the form by clicking on the link below:

Plant Physiology First Author Feature Form: https://forms.office.com/r/ZBCGZfhm8F
If you cannot access the form through the link above, please contact Rachel at rbelsky@aspb.org.

To upload an image of yourself to accompany your profile, please put your photo in the correct journal folder at the link below. You must label your image: Author First Name Last Name Article Title.

First Author Profile Image Dropbox: https://www.dropbox.com/scl/fo/xz029p7qjr2jml2rp2kmq/h?dl=0&rlkey=6fkxcaqmxx35ap152b2osy1fz

=====
IMPORTANT REMINDER: PEER REVIEW REPORTS

=====

If you opted to publish a peer review report along with your article during the original submission process, it will be prepared by the editorial staff and publicly posted with your manuscript, inside the zip file that contains any other supplemental material. As a reminder, the peer review report is a public record of all comments from editors and reviewers, as well as your prior responses, as you received them in the decision letters for each draft of your manuscript. If you agreed to publish this report and have changed your mind, or are not sure if you selected this option, please contact the editorial office as soon as possible before signing the license agreement from our publisher.

-------------------------------------------------------------------------- Reviewer comments:
